# A low-light image enhancement method with brightness balance and detail preservation

**DOI:** 10.1371/journal.pone.0262478

**Published:** 2022-05-31

**Authors:** Canlin Li, Jinjuan Zhu, Lihua Bi, Weizheng Zhang, Yan Liu

**Affiliations:** School of Computer and Communication Engineering, Zhengzhou University of Light Industry, Zhengzhou, China; University of Engineering & Technology, Taxila, PAKISTAN

## Abstract

This paper proposes a new method for low-light image enhancement with balancing image brightness and preserving image details, this method can improve the brightness and contrast of low-light images while maintaining image details. Traditional histogram equalization methods often lead to excessive enhancement and loss of details, thereby resulting in an unclear and unnatural appearance. In this method, the image is processed bidirectionally. On the one hand, the image is processed by double histogram equalization with double automatic platform method based on improved cuckoo search (CS) algorithm, where the image histogram is segmented firstly, and the platform limit is selected according to the histogram statistics and improved CS technology. Then, the sub-histograms are clipped by two platforms and carried out the histogram equalization respectively. Finally, an image with balanced brightness and good contrast can be obtained. On the other hand, the main structure of the image is extracted based on the total variation model, and the image mask with all the texture details is made by removing the main structure of the image. Eventually, the final enhanced image is obtained by adding the mask with texture details to the image with balanced brightness and good contrast. Compared with the existing methods, the proposed algorithm significantly enhances the visual effect of the low-light images, based on human subjective evaluation and objective evaluation indices. Experimental results show that the proposed method in this paper is better than the existing methods.

## 1 Introduction

People often get some poor quality images in the process of image acquisition because of the complexity of the geographical environment and time. For example, the low-light image often has shortcomings of low contrast, uneven brightness and poor image visibility due to the lacking of light source, which is extremely unfavorable for the subsequent processing of images such as feature extraction and image segmentation. However, low-light images are often produced in real life. The general causes of low light can be divided into the following situations roughly: (1) In the night, the images are very dark due to insufficient or no light source; (2) The uneven illumination caused by the shading of buildings leads to the existence of dark regions in the image; (3) There are also in special places, such as underground mines, interiors and other dark places. People are also difficult to capture satisfactory images. It can be seen that this problem has affected the daily life of people including photography, forensics, traffic monitoring and even engineering safety monitoring [1]. Therefore, it has become a challenging and very important task to enhance the low-light image [2]. The purpose of the low-light image enhancement is to improve the brightness and clarity of the image so that people can obtain more useful and accurate information from it. In recent years, this issue has become a hot research topic, many scholars have studied and discussed it.

In the past few years, a variety of image enhancement methods have been proposed. For the low-light image enhancement, many classical algorithms have been proposed, such as histogram equalization (HE) [3], gamma correction [4], retinex theory [5], etc. HE algorithms mainly focus on enhancing image contrast. HE method has received considerable attention due to its simple and direct implementation. It mainly remaps the image gray level, so that the histogram of the image follows the uniform distribution [6]. However, the traditional HE methods use the same histogram transformation for the whole image, and do not take into account the characteristics of special images. For example, the enhancement effect of this method is not significant when there are uneven brightness areas in the image. For low illumination image, due to its low dynamic range (low contrast), the image quality will be reduced in the process of histogram equalization and single gray level change. In order to solve this problem, different versions of improved methods have appeared. Considering the difference of the image local contrast, some scholars proposed adaptive histogram equalization (AHE) [7]. AHE algorithm performs histogram enhancement for each pixel by calculating the transformation function of each pixel’s neighborhood. AHE algorithm takes into account the local information of the image and effectively improves the image local contrast. However, its disadvantage is that it enlarges the noise in the homogeneous region of the image while improving the contrast. Therefore, the contrast limited adaptive histogram equalization (CLAHE) algorithm [8] is proposed. Based on AHE algorithm, CLAHE algorithm limits the histogram of each sub block, which can control the noise caused by AHE algorithm and make the image contrast more natural. In addition, in order to overcome the brightness problem, researchers have proposed some methods to maintain the average brightness based on HE algorithm. For example, the double histogram equalization method based on image mean segmentation [9] advocates that the average brightness of the input image can be maintained while the contrast of the enhanced image is low. However, the enhancement effect of this method for low illumination image in extremely dark environment is not ideal, and there is a strong oversaturation phenomenon. Similarly, recursive mean segmentation histogram equalization (RMSHE) [10] and brightness-preserving dynamic histogram equalization (BPDHE) [11] can keep the average brightness of the input image when processing the image output, but they may still not generate images with ideal natural appearance. In 2012, Khan et al. proposed an algorithm based on brightness maintenance, namely weighted average multi-segment histogram equalization (WAMSHE) [12]. Unfortunately, this method often has excessive enhancement when processing some images, due to the pixel ratio of the image segmentation regions is too large. Using the platform-based clipping method to modify the histogram can avoid intensity saturation and improve excessive enhancement. Ooi et al. [13] proposed a platform constrained double histogram equalization (BHEPL) method in 2009. Firstly, the input histogram is segmented according to the average value of the original histogram, and then the sub histogram is clipped by using the platform limit value, and then the traditional processing is performed. In addition, there are quadrant dynamic histogram equalization (QDHE) [14] and dynamic quadrant histogram equalization platform limitation (DQHEPL) [15], DQHEPL is an improvement of BHEPL, which divides all images into four sub images for equalization operation, so it can’t keep the stability of gray level for some images. The histogram-based method has achieved good results in enhancing image contrast, but often overlooked the image details. Gamma correction is also based on the grayscale transformation of the image to achieve the improvement of brightness, and the effect of detail restoration is not outstanding [16]. Retinex theory advocates maintaining the color constancy of the image [17], which can retain the original details of the image, but it is relatively weak in balancing the brightness of the image, and halos and artifacts often appear [18].Classical algorithms based on retinex theory include single-scale retinex (SSR) algorithm [19], multi-scale retinex (MSR) algorithm [20], multi-scale retinex with colour restore (MSRCR) [21], and so on. The low-light image enhancement via illumination map estimation (LIME) method proposed by X Guo et al. [22] is also based on the retinex theory, but this method refine the initial illumination map by imposing a structure prior on it, as the final illumination map, so it can get better enhancement effect.

For the enhancement of low-light images, maintaining image details and improving image contrast are theoretically opposed, and it is difficult to keep a balance between the two in the same algorithm. In order to achieve the purpose of improving image contrast and retaining image details at the same time, some new methods have emerged. To achieve the trade-off among detail enhancement, local contrast improvement and maintaining the natural feeling of the image, reference [23] used weighted fusion strategy to weigh the advantages of different technologies. In reference [24], the image is divided into structure layer and texture layer for processing, so as to preserve the image details. Reference [25] uses discrete wavelet transform (DWT) to decompose the image into detail coefficients containing edge information (image details) and approximate coefficients containing illumination information, and the approximate coefficients are separately enhanced to protect image details. In addition, there are some new methods based on intelligent optimization algorithms, such as genetic algorithm (GA) [26], particle swarm optimization (PSO) algorithm [27], artificial bee colony (ABC) algorithm [28] and cuckoo search (CS) algorithm [29]. Reference [30] introduces the method of using GA and PSO algorithm to enhance contrast in spatial domain and frequency domain. Due to the introduction of GA and PSO algorithm, good image results are obtained. The introduction of such optimization algorithms can help to solve many complex problems in image processing. Reference [31] regards the enhancement of gray image contrast as an optimization problem, which is solved by combining PSO and discrete wavelet transform (DWT). By adjusting a new expansion parameter, the target fitness criterion is maximized to enhance the contrast and local details of the image. Reference [32] proposed an adaptive image enhancement method based on non-subsampled contour transformation (NSCT), fuzzy sets and ABC optimization, which helps to improve the low contrast and low clarity of images obtained in practical applications, this method optimizes fuzzy parameters through ABC algorithm, which improves its adaptability. In reference [33], a channel segmentation image enhancement method based on discrete shearlet transform and PSO algorithm is proposed. By using PSO algorithm, image artifacts and unnatural forces caused by high directional coefficients are eliminated, and the brightness component of the image is enhanced. Reference [34] firstly applied the sigmoid function to enhance the image when processing remote sensing images, and then adopted a multi-objective particle swarm optimization algorithm to maximize the amount of information in the image while maintaining image intensity. Experiments show that this method can retain significant details on the original image. Swarm intelligence optimization algorithm has made remarkable achievements in improving the adaptability of image enhancement algorithm and simplifying the complex problems in image enhancement.

In summary, in the issue of low-light image enhancement, the fusion strategy is effective in theory to improve the image contrast and preserve the image details at the same time. The existing methods can be roughly divided into two ideas: One is to separate the original image details by transforming the space to achieve the effect of detail preservation. In this method, few people consider that there will be noises in the detail components, and at the same time, there may be noise amplification problems during the inverse transformation process, which resulting in unsatisfactory results. The other is to achieve the goal by weighted fusion of images with different advantages processed by each method, but the adaptability of this model is relatively weak and can’t be applied to different natural images. The results of image processing with different features may vary greatly. The method proposed in this paper is similar to the image fusion strategy, but it does not adopt the weighted fusion method. The purpose of this paper is to improve the image contrast and balance the image brightness while preserving the details of the original image. For improving the contrast of the image and enhancing the brightness of the image, HE method has been very outstanding. In this paper, the double histogram equalization with a double automatic platform (DHEDAPL) method based on improved CS algorithm is used to process the image, we can obtain the image A with balanced brightness and good contrast. In the reference [35], the author proposed the method named bi-histogram equalization using two plateau limits (BHE2PL), but plateau limits is only calculated by the statistics of histograms in this method, a single platform calculation method cannot maximize the advantages of this method. In this paper, we get the range of platform limit through histogram statistics, and use the improved CS algorithm to select optimal value in the range. In view of the aspect of retaining image details, this paper uses the method based on total variation in reference [36], to extract the main structure of the image, and then get the texture detail mask B which contains all the texture details of the image, this method can obtain the image details by extracting the image texture, and has good noise resistance, and at the same time, we don’t need the inverse transformation process, so the problem of noise amplification is avoided. Finally, we add texture detail mask B directly to image A to obtain the final enhanced image C.

The structure of the rest of this paper is as follows: The second part introduces the related work in this paper; The third part describes the algorithm framework and implementation steps in detail; The fourth part conducts experiments on the method proposed in this paper, firstly, it verifies the anti-noise performance of the proposed model, then compares the method in this paper with other mainstream algorithms, and analyzes it through two aspects: human subjective evaluation and objective image quality indicators; The last chapter summarizes the work of this paper.

## 2 Related theories

Through the analysis of the introduction of the previous chapter, we know that in recent years, more and more scholars try to transform image problems into optimization problems. Therefore, the application of intelligent optimization algorithms in image processing is becoming more and more mature, including the PSO algorithm and the CS algorithm are frequently used, both of which have their own advantages, but also have their own limitations. The HE method is simple, has certain advantages in enhancing image contrast and brightness, and is also favored by many scholars. However, the problems of over enhancement and loss of image details of traditional histogram have always been concerned by scholars and urgently need to be solved. This paper designs a method for low-light image enhancement with balancing image brightness and preserving image details, which aims to solve the above problems. In this chapter, we mainly introduce three classical theories: particle swarm optimization algorithm, cuckoo search algorithm and histogram equalization algorithm, it paves the way for the next chapter to introduce the design method of this paper.

### 2.1 PSO algorithm

The particle swarm optimization (PSO) algorithm was originally an evolutionary algorithm proposed by James Kennedy and Russell Eberhart in 1995 [27]. Its development is based on the study of the social behavior of bird predation. The basic idea of the standard particle swarm optimization algorithm is: assuming that the search space is M-dimensional and there are N particles in the space, the spatial position of the *i*-th particle can be expressed as $X_{i}=(x_{i1},x_{i2},\ldots ,x_{iM}),i=1,2,\ldots ,N,$ and velocity can be expressed as $V_{i}=(v_{i1},v_{i2},\ldots ,v_{iM})$. Defining the historical best advantage of the particle (Personal best) is pbest, denoted as $P_{i}=(p_{i1},p_{i2},\ldots ,p_{iM})$, the global best found by defining the population is gbest, denoted as $P_{g}=(p_{g1},p_{g2},\ldots ,p_{gM})$. The first step of PSO algorithm is to initialize the population particles as random particles (random solutions), and then find the optimal solution iteratively. In each iteration, the particles update their speed and position through two extreme values pbest and gbest. For particles in the *m*-th (1≤*m*≤*M*) dimension, the iterative update formula is as follows:

$$V_{im}(t+1)=\omega V_{im}(t)+c_{1}r_{1}(P_{im}(t)-X_{im}(t))+c_{2}r_{2}(P_{gm}(t)-X_{im}(t))$$ (1)

$$X_{im}(t+1)=X_{im}(t)+V_{im}(t+1)$$ (2)

**Fig 1:**
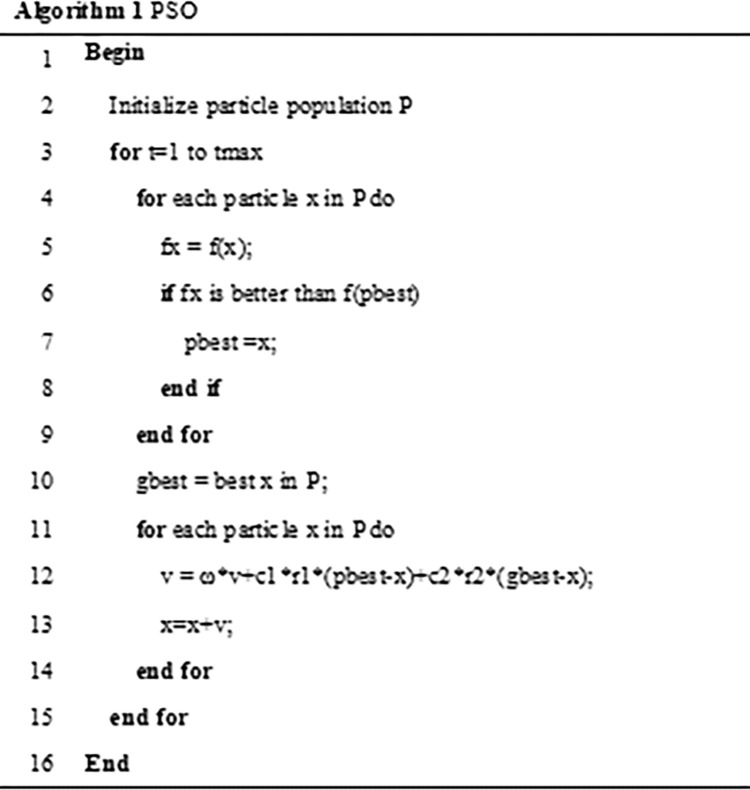
PSO.

Where the parameter *ω* in Formula (1) is the inertia weight value, c_1_ and c_2_ are learning factors, *r*_1_ and *r*_2_ are random numbers in the range of [0, 1]. The update of the particle state depends on three aspects: The first is the inheritance of the previous particle speed based on the trust of particle in the previous own state, which belongs to the "inherited part". The second is the learning of itself, which is an extension of itself. The process of the state belongs to the "cognitive part". The third is the learning of the group, which reflects the mutual sharing and cooperation of information among particles and belongs to the "social part". The pseudo code of PSO algorithm is shown in Fig 1.

The PSO algorithm is widely introduced into the application of image enhancement field because of its simple principle and easy implementation, but it is easy to fall into the local optimum and the search accuracy is not enough.

### 2.2 CS algorithm

In 2009, Yang and Deb developed the algorithm based on the parasitic reproduction behavior of cuckoos [29]. The cuckoo search (CS) algorithm mainly simulates the parasitic cuckoo’s nestling and the flight mechanism of certain birds [37] to effectively solve the optimization problem. The cuckoo relies on the nests of other hosts to lay eggs. The host bird treats the eggs as its own eggs. If the host bird recognizes the foreign eggs, the host bird will either throw away the eggs or leave the nest and build a new nest at a new location. Assuming that there is a bird egg in each nest, each bird egg represents a solution, and the new solution is represented by the bird egg. The basic goal of the CS algorithm is to find the best nest to incubate the bird egg by random walk. The three idealized rules to be considered to form a theory of cuckoo’s nest finding are:

1. Each cuckoo lays one egg at a time and then places it in a randomly selected nest.
2. In a randomly selected set of nests, those nests with high-quality eggs will be carried over to the next generation.
3. The number of available host nests is fixed, and the probability that the eggs laid by the cuckoo will be found by the host bird is *Pa*∈[0, 1].

Based on the above three idealized rules, the cuckoo optimization search uses Formula (3) to update the position of the next generation nest:

$$x_{i}^{\left(t+1\right)}=x_{i}^{\left(t\right)}+\alpha ⊕Levy(\lambda ),\;(i=1,2,\ldots n)$$ (3)

Where $x_{i}^{\left(t\right)}$ represents the *i*-th nest-position in the *t*-th generation, ⊕ represents the point-to-point multiplication, and *α* represents the step size factor, which is used to control the step size, and the value is usually set to 1. *Levy*(*λ*) is a random search path generated by the *Levy* flight that obeys the parameter *λ*, and its moving step length obeys the stable distribution of *Levy*:

$$Levy∼\mu =t^{-\lambda },\;1<\lambda \leq 3$$ (4)

**Fig 2:**
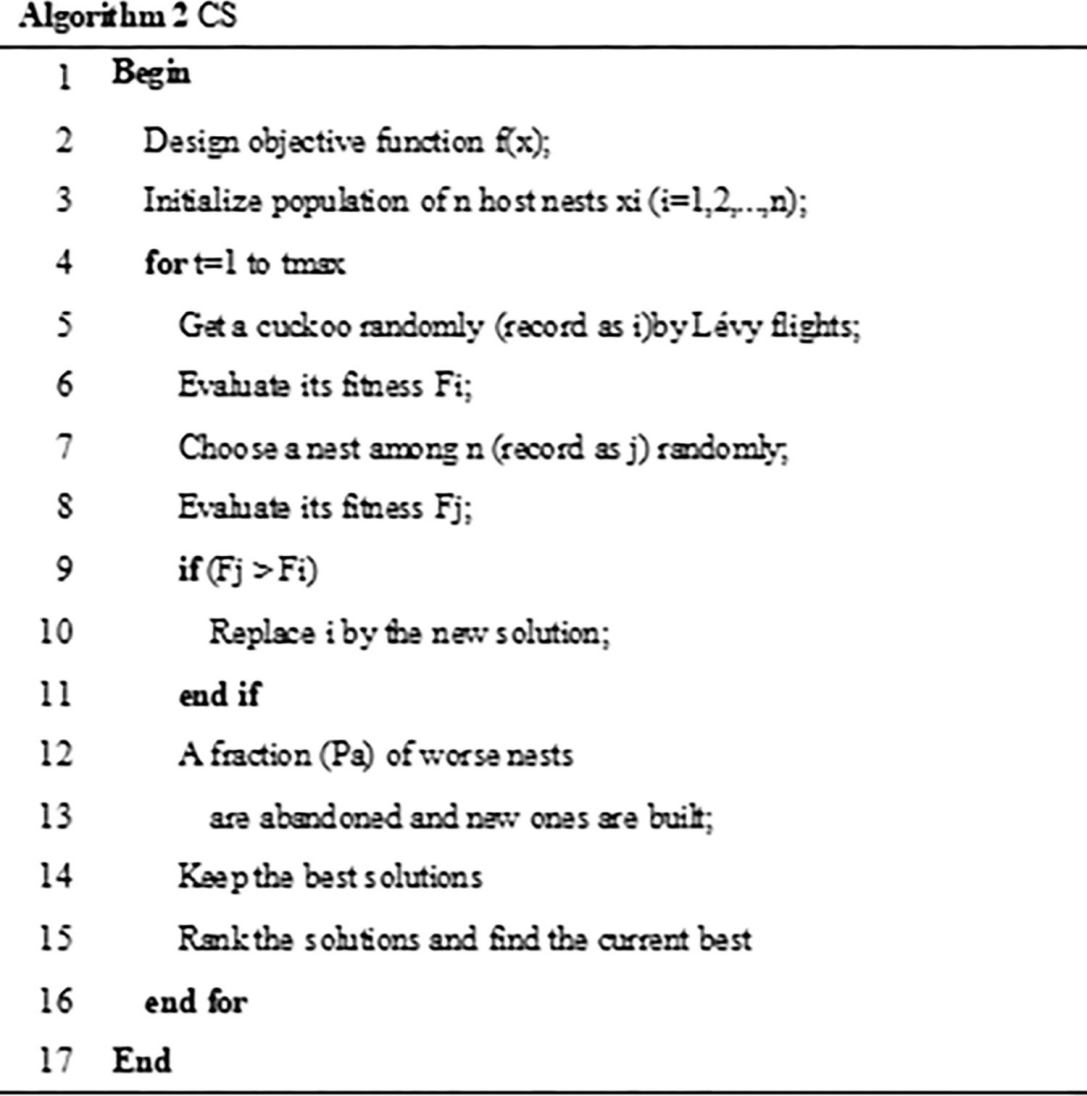
CS.

Where *μ* obeys the normal distribution, *λ* is the power coefficient, and *λ* = 1.5. It can be seen from Formula (4) that the optimization path of the CS algorithm is composed of two parts, namely frequent short jumps and occasional long jumps. This optimization method can make it easier for the algorithm to jump out of the local optimum. The pseudo code of CS algorithm is shown in Fig 2.

### 2.3 Histogram equalization

The histogram equalization (HE) algorithm [6] is a method of automatically adjusting image contrast using grayscale transformation, it calculates the gray scale transformation function by the probability density function of gray scale. It is a histogram correction method based on the cumulative distribution function transformation method. For an image *X* of size *M*×*N*, take (*i*,*j*) as the spatial coordinates of the pixels in the image, and *X*(*i*,*j*) represents the intensity of pixels in the image. The pixel value range of each coordinate is *k* = 0,1,…,*L*−1, where *L* is the maximum gray level in the image. Then the histogram *H* associated with the image describing the frequency of the intensity value in the image is defined as:

$$H(k)=n_{k}$$ (5)

Where *n*_*k*_ represents the number of occurrences of intensity k in the image. The probability density function *p*(*k*) related to *H* is defined as:

$$p(k)=\frac{H(k)}{M\times N}$$ (6)

Where *p*(*k*) represents the probability of intensity k. Then its cumulative density function *c*(*k*) is:

$$c(k)=\sum_{i=X_{0}}^{k}p(i)$$ (7)

Where *X*_0_ is the lowest intensity within the range for calculating the cumulative density function, the transformation function *f*(*k*) associated with standard histogram equalization uses *c*(*k*) to map the input image to the dynamic range [*X*_0_, *X*_*L*−1_]. For the special case of HE, *X*_0_ = 0, *X*_*L*−1_ = *L*−1, the mapping function is as follows:

$$f(k)=X_{0}+(X_{L-1}-X_{0})\times c(k)$$ (8)

Then the result image *Y* = {*Y*(*i*,*j*)} produced by the histogram equalization can be expressed as:

Y={Y(i,j)}={f(X(i,j))|∀X(i,j)∈X} (9)

HE algorithm expands the dynamic range of the histogram, but at the same time, the gray level of the transformed image is reduced, which will cause the loss of some details. For some images with peaks, the processing of HE algorithm can cause unnatural effects of excessive enhancement.

## 3 The proposed method

### 3.1 Framework description

**Fig 3:**
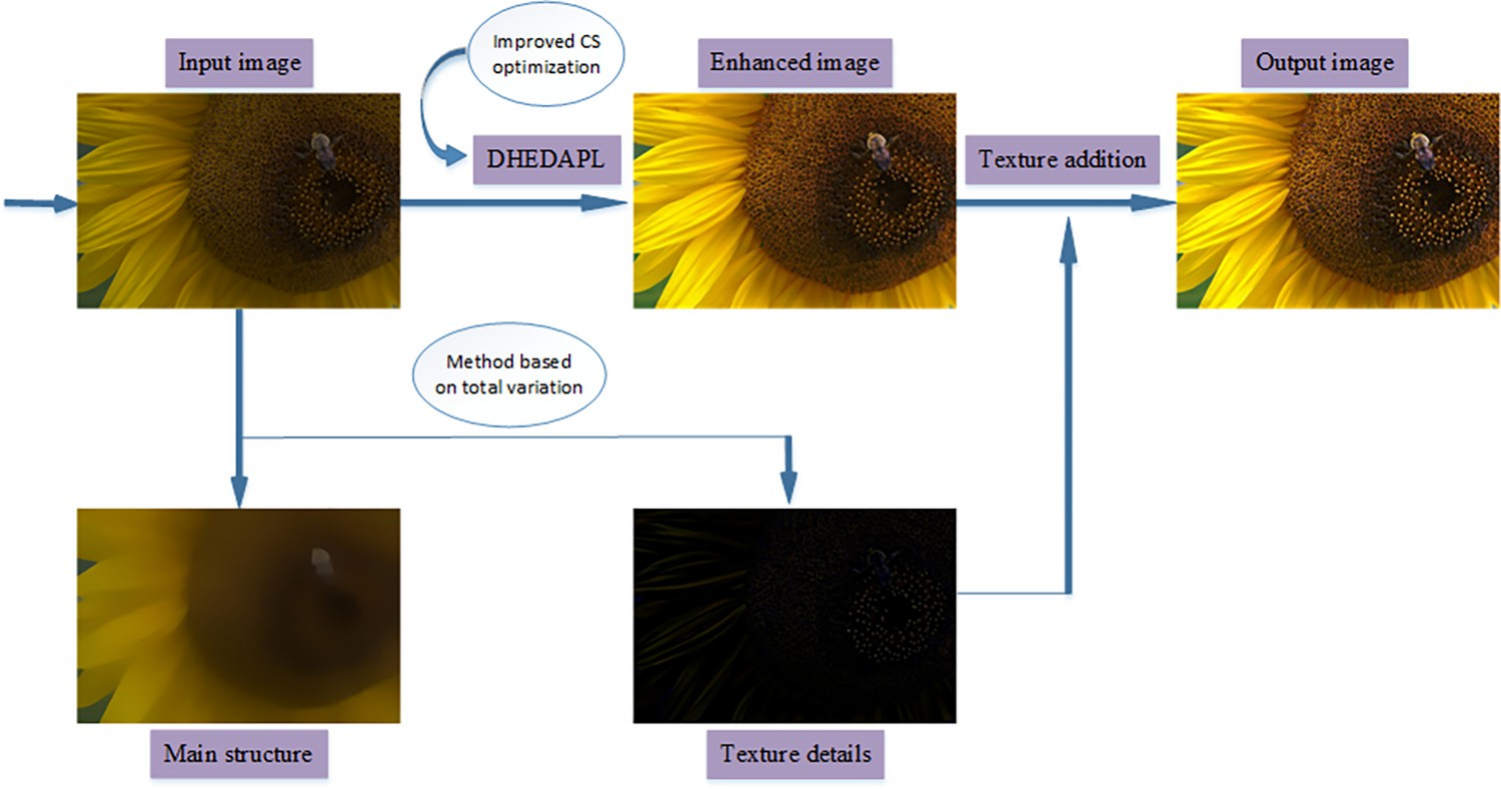
The flow chart of the image processing method proposed in this article.

Starting from the characteristics of low-light images, the proposed method aims to improve the contrast of low-light images, balance its brightness while retaining its detailed features. In order to obtain an image with good contrast and balanced image brightness, this paper uses the DHEDAPL method based on improved CS algorithm. First, the image histogram is segmented, and then the histogram statistical information and improved CS algorithm are used to determine the limit value of each sub-histogram platform, use the corresponding platform limit to clip each sub-histogram separately, and after obtaining the corrected histogram, perform traditional histogram equalization on the sub-histogram. Here the histogram of the original image is divided into two, there are four platform limits and eight corresponding limit values. The improved CS optimization method is used here, each platform value can be selected within a certain range, and we select the optimal value through the evaluation function. This method is more adaptive than other methods that calculate the platform limit in a fixed way. The image detail texture feature is a kind of global feature, which is different from the color feature. The texture feature is not based on the feature of the pixel, but needs to be statistically calculated in the area containing multiple pixels. An image can use "structure + Texture" to express, but usually extracting the image structure will emphasize the regularity of the texture. We use the method based on the total variation model to extract the main structure of the image. The model does not require texture rules or symmetry, and is general and random. By removing the main structure of the image, we can get the texture that contains all the details of the image. An image can be expressed in the form of "structure + texture", but the regularity of the texture is usually emphasized when extracting the image structure. We use the method based on the total variation model to extract the main structure of the image, which does not require texture rules or symmetrical, it is general and random. By removing the main structure of the image, we can get the texture that contains all the details of the image. Different from the image detail layer extracted by other transformations, the image detail obtained by texture extraction has good noise resistance. Finally, we add the texture to the image processed by DHEDAPL optimized based on improved CS algorithm to obtain the final enhanced image. As shown in Fig 3, it is a main schematic of low-light image enhancement. Next, we will introduce the specific steps of each link in detail.

### 3.2 Improved CS algorithm

**Fig 4:**
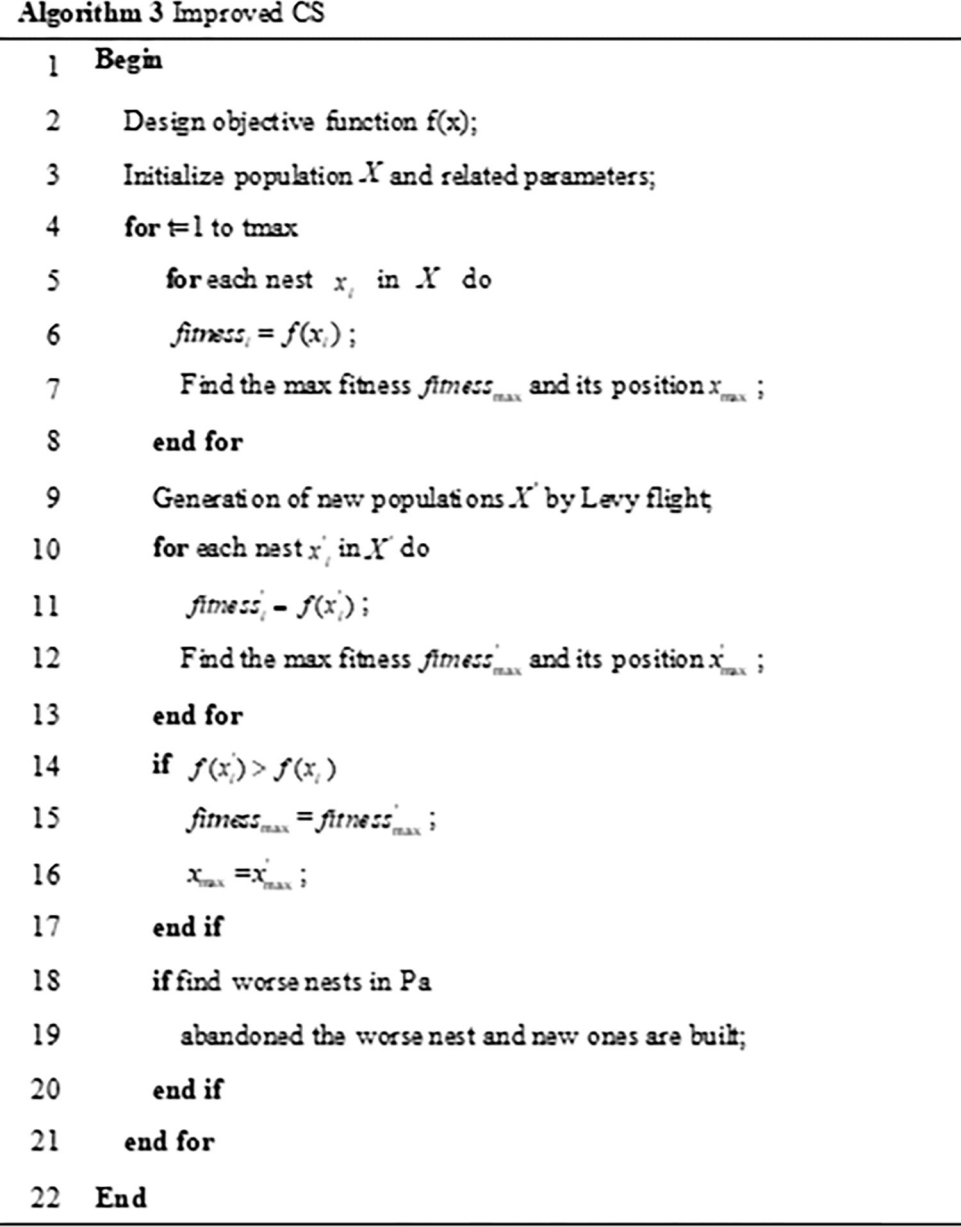
Improved CS.

**Fig 5:**
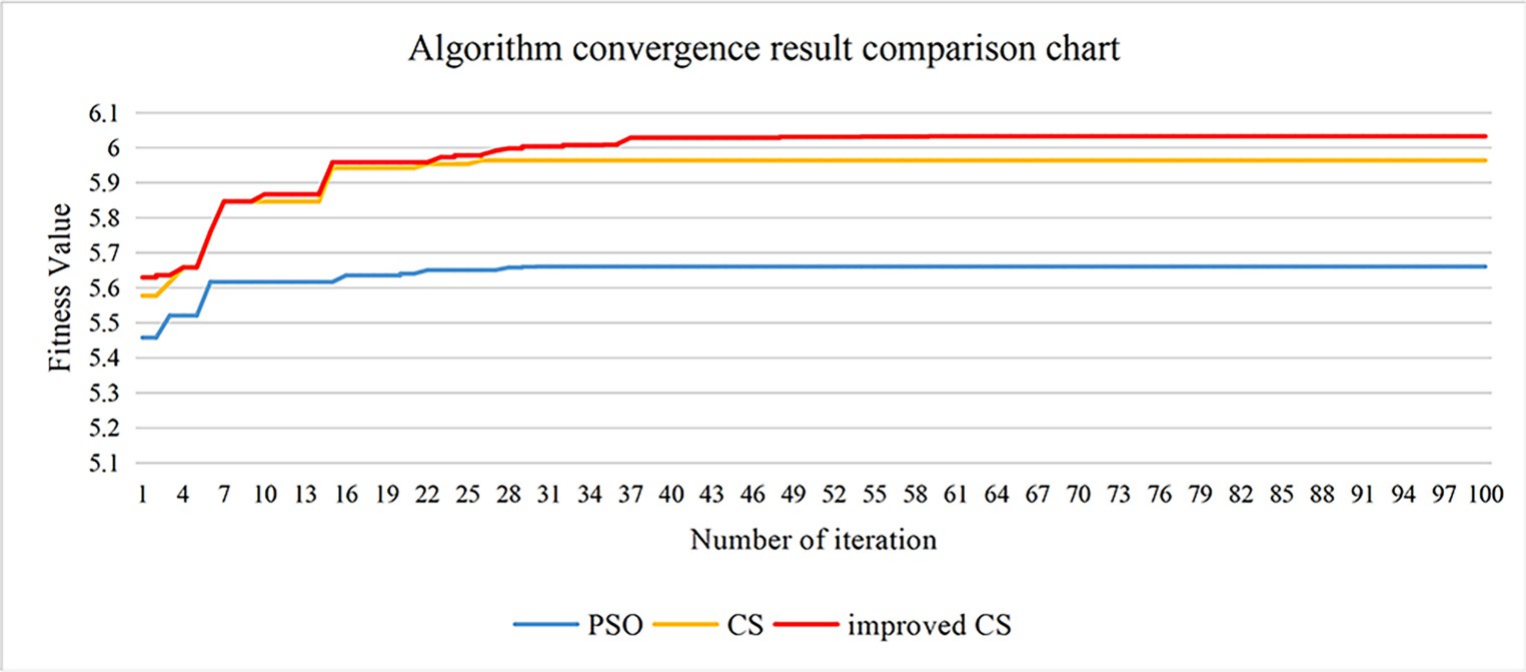
Convergence rate analysis for platform limit parameter optimization using PSO, CS and improved CS.

In the subsection 2.1 and subsection 2.2 of chapter 2, the standard PSO algorithm and CS algorithm have been introduced. At present, these two swarm intelligence optimization algorithms are widely used in various fields and have been verified in the direction of image enhancement, they are all effective optimization methods. However, the individual evaluation of the two algorithms shows certain shortcomings. The most obvious feature of the PSO algorithm is that it is easy to fall into the local optimum. In addition, CS algorithm uses frequent short jumps and occasional long jumps in the optimization process, this optimization method can make it easier to jump out of the local optimum for the algorithm, at the same time, CS algorithm also has the disadvantages of slowing convergence speed and lacking of vitality in later search [38], the reason is that the CS algorithm uses random selection when updating the population, so that the local update can’t quickly find the truly optimal nest position. Inspired by PSO algorithm, it is easier to find the best nest by traversing the population position. Under the framework of the CS algorithm, improved CS algorithm uses the traversal idea of PSO algorithm to design a new optimization search method. This method is easy to jump out of the local optimum and converges quickly. Its algorithm flow is shown in the Fig 4. Then, we conducted a comparative experiment of improved CS algorithm, PSO algorithm, and CS algorithm, the results are shown in Fig 5, It can be seen that the convergence speed of improved CS algorithm is obviously better than that of PSO algorithm and CS algorithm, the particles can still maintain the exploration vitality in the middle and later stage, and the optimization result is also better than that of PSO algorithm and CS algorithm.

### 3.3 Double histogram equalization with double automatic platform

As shown in subsection 3.1, we first perform DHEDAPL processing on the image, in the new equalization method proposed here, the original histogram is first divided to obtain two sub-histograms. Here, four platform limits are used, and each sub-histogram uses two limit values. Different from other methods, the method in this paper is no longer just based on the statistics of the histogram for the selection of the platform limit value, but for each platform limit value according to the histogram statistical information to set the upper and lower bounds, and then use the improved CS algorithm to wandering evaluation within the range, the selection of the platform limit value will be selected within this range adaptively.

**Fig 6:**
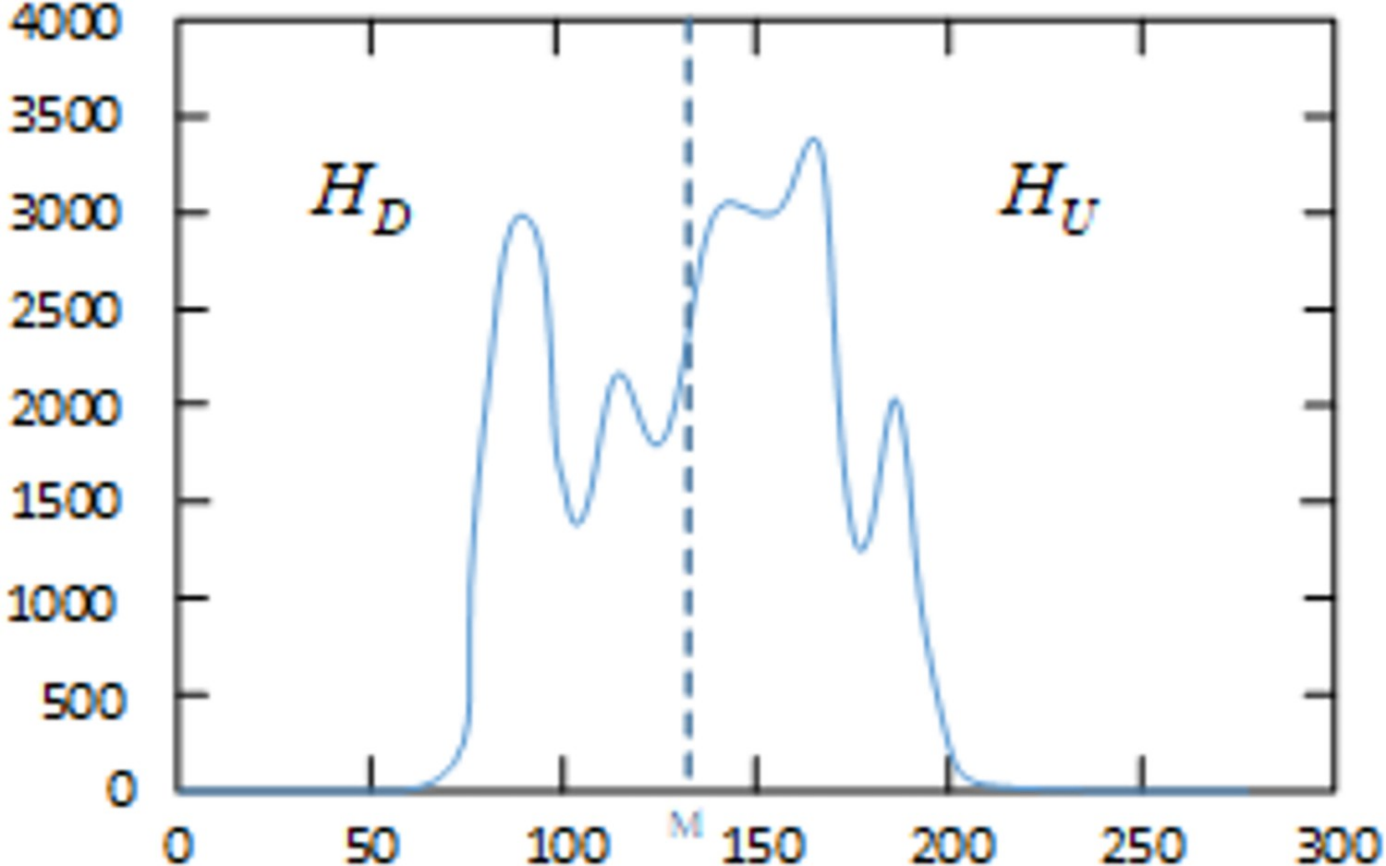
Histogram segmentation.

First, we calculate the average intensity *M* of the global histogram, and clip the histogram according to the average intensity *M* (as shown in Fig 6). The calculation formula for *M* is as follows:

$$M=\sum_{k=0}^{L-1}p(k)\times k$$ (10)

Where *p*(*k*) is the probability of intensity k, its calculation method is shown in Formula (6).

After calculating the value of *M*, the original histogram is divided into obtain two sub-histograms, which are the lower histogram *H*_*D*_ and the upper histogram *H*_*U*_. *H*_*D*_ contains all the intensity values between [*I*_min_, *M*], and *H*_*U*_ contains All intensity values between [*M*+1, *I*_max_], where *I*_min_ represents the minimum intensity value that has appeared at least once in the image, and *I*_max_ represents the maximum intensity value that appeared in the image.

After segmenting the global histogram, calculating the platform limit L of each sub-histogram is the next task. The classic platform value calculation method is as follows:

L=C×Pk (11)

In the formula, *C* is a coefficient whose value is between 0 and 1, and *Pk* is the peak value in the histogram, that is:

Pk=max{H(k)|k=0,…,L−1} (12)

This article will use the local information obtained from the input histogram to predetermine the range of the platform limit *L*. Here, the gray scale ratio *GC* of the sub-histogram is calculated instead of C in the formula, and *GC* is used as a coefficient to calculate the basic value of each platform limit:

$$L_{D1}=GC_{D1}\times Pk_{D}$$ (13)

$$L_{D2}=GC_{D2}\times Pk_{D}$$ (14)

$$L_{U1}=GC_{U1}\times Pk_{U}$$ (15)

$$L_{U2}=GC_{U2}\times Pk_{U}$$ (16)

Where *Pk*_*D*_ and *Pk*_*U*_ are the maximum intensity peaks of the lower sub-histogram and the upper sub-histogram respectively, *L*_*D*1_ and *L*_*D*2_ are the base values of the lower and upper platform for lower sub-histograms, *L*_*U*1_ and *L*_*U*2_ are the base values of the lower and upper platform for upper sub-histogram. The gray scale ratios *GC*_*D*1_ and *GC*_*D*2_ of the lower sub-histogram and the gray scale ratios *GC*_*U*1_ and *GC*_*U*2_ of the upper sub-histogram are defined as:

$$GC_{D1}=\frac{M-M_{D}}{M-I_{\min}}$$ (17)

$$GC_{D2}=GC_{D1}+\lambda_{D}$$ (18)

$$GC_{U1}=\frac{I_{\max}-M_{U}}{I_{\max}-M}$$ (19)

$$GC_{U2}=GC_{U1}+\lambda_{U}$$ (20)

Where *M*_*D*_ and *M*_*U*_ are the average intensities of the lower sub-histogram and the upper sub-histogram respectively, *λ*_*D*_ and *λ*_*U*_ are the gray scale ratios differences of the lower sub-histogram and the upper sub-histogram respectively. The calculation method is as follows:

$$M_{D}=\frac{\sum_{k=I_{\min}}^{M}k\times H(k)}{N_{D}}$$ (21)

$$M_{U}=\frac{\sum_{k=M+1}^{I_{\max}}k\times H(k)}{N_{U}}$$ (22)

$$\lambda_{D}=\{\begin{array}{cc} \frac{1-GC_{D1}}{2}, & \mathrm{if}\;GC_{D1}>0.5\\ \frac{GC_{D1}}{2}, & \mathrm{if}\;GC_{D1}\leq 0.5 \end{array}$$ (23)

$$\lambda_{U}=\{\begin{array}{cc} \frac{1-GC_{U1}}{2}, & \mathrm{if}\;GC_{U1}>0.5\\ \frac{GC_{U1}}{2}, & \mathrm{if}\;GC_{U1}\leq 0.5 \end{array}$$ (24)

Where *N*_*D*_ and *N*_*U*_ are the total number of pixels in the lower sub-histogram and the upper sub-histogram respectively.

At this time, the basic values *L*_*D*1_, *L*_*D*2_, *L*_*U*1_, and *L*_*U*2_ of each platform have been obtained, and finally we set the range of each platform value as follows:

$$\frac{L_{D1}}{4}<L_{D1}^{'}\leq \frac{L_{D1}+L_{D2}}{2}$$ (25)

$$\frac{L_{D1}+L_{D2}}{2}<L_{D2}^{'}\leq Pk_{D}$$ (26)

$$\frac{L_{U1}}{4}<L_{U1}^{'}\leq \frac{L_{U1}+L_{U2}}{2}$$ (27)

$$\frac{L_{U1}+L_{U2}}{2}<L_{U2}^{'}\leq Pk_{U}$$ (28)

Within this range, improved CS is used to optimize the value selection to obtain the final platform limit values $L_{D1}^{'}{}_{}$, $L_{D2}^{'}$, $L_{U1}^{'}{}_{}$, and $L_{U2}^{'}{}_{}$, and then clip the histogram. The application of improved CS in this method will be described in detail in next section. For the value of the lower sub-histogram (*I*_min_<*k*≤*M*) that is less than or equal to $L_{D1}^{'}{}_{}$, use the value of $L_{D1}^{'}{}_{}$ to modify the sub-histogram, if the value is greater than the value of $L_{D2}^{'}$, use the value of $L_{D2}^{'}$ to modify the histogram. Similarly, for the value of the upper sub-histogram (*M*+1<*k*≤*I*_max_) that is less than or equal to $L_{U1}^{'}{}_{}$, use the value of $L_{U1}^{'}{}_{}$ to modify the sub-histogram, if the value is greater than the value of $L_{U2}^{'}{}_{}$, use the value of $L_{U2}^{'}{}_{}$ to modify the histogram. The specific rules are as follows:

$$H_{D}(k)=\{\begin{array}{ccc} L_{D1}^{'}, & \mathrm{if} & H_{D}(k)\leq L_{D1}^{'}\\ H_{D}(k), & \mathrm{if} & L_{D1}^{'}<H_{D}(k)\leq L_{D2}^{'}\\ L_{D2}^{'}, & \mathrm{if} & H_{D}(k)>L_{D2}^{'} \end{array}$$ (29)

$$H_{U}(k)=\{\begin{array}{ccc} L_{U1}, & \mathrm{if} & H_{U}(k)\leq L_{U1}\\ H_{U}(k), & \mathrm{if} & L_{U1}<H_{U}(k)\leq PL_{U2}\\ L_{U2}, & \mathrm{if} & H_{U}(k)>PL_{U2} \end{array}$$ (30)

**Fig 7:**
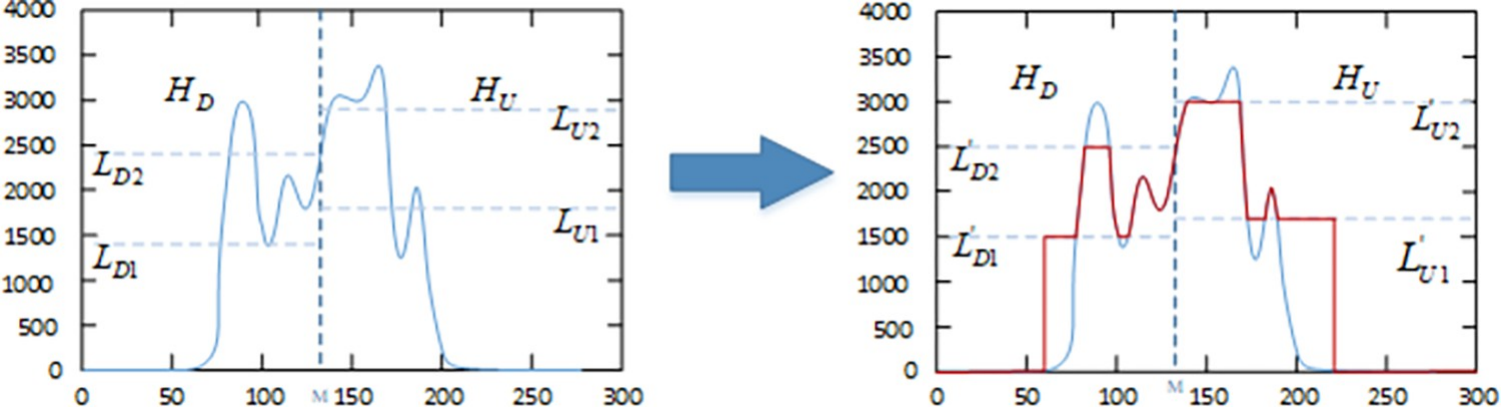
Histogram clipping.

The process of modifying the histogram is shown in Fig 7. After the histogram is modified, the sub-histogram can be independently equalized according to Formula (9) to obtain an enhanced image. This process can be expressed by the following formula:

$$I_{E}=\Gamma (I)$$ (31)

Where *I* represents the original input image, Γ represents the process of dual-histogram dual-adaptive platform processing, and *I*_*E*_ represents the output image after our enhancement processing.

### 3.4 Optimization of platform value with improved CS

As mentioned in the subsection 3.3, the method to obtain the basic range of each platform value has been determined. After obtaining the range, we use improved CS to explore the space range and judge the quality of the platform value through the evaluation function, the platform value optimization process can be divided into the following steps:

Step 1: Initialize the number of nests N and dimension D, initialize the nest position *X* within the basic range of the platform, find probability Pa, the maximum number of iterations T and the fitness value set *fitness*;

Step 2: Traverse the position *X*(*i*,:) in the population, use the parameters of the corresponding position as the platform value, perform DHEDAPL enhancement on the image, evaluate the corresponding enhanced image using the evaluation function, store the corresponding fitness value *fitness*_*i*_, compare it to get the largest fitness value *fitness*_max_ and save the position of the nest as *X*(max,:);

Step 3: Update the population through Lévy flight to obtain a new population *X*’, traverse the population position *X*’(*i*,:), repeat the operation of step 2 to obtain the corresponding image enhancement result and fitness value $fitness_{i}{}^{'}$, as well as the corresponding maximum fitness value $fitness_{\max}^{'}{}_{}$ and its position *X*’(max,:);

Step 4: Determine whether to perform population replacement. If $fitness_{\max}<fitness_{\max}^{'}$, replace the original population with the population updated by Lévy flight, and update the maximum fitness value *fitness*_max_ and position *X*(max,:) at the same time;

Step 5: Determine whether the cuckoo egg is found, if it is found, update the population by Formulas (1) (2), if it is not found, keep the nest position.

Step 6: Judge whether the iteration stop condition is reached, if not, then jump to Step2, if the iteration stop condition is met, exit the loop. The last retained position with the maximum fitness value carries the parameters as the final DHEDAPL corresponding platform value.

Where the fitness function, as a criterion for judging the quality of the location, is the key to the effectiveness of the final algorithm. The design of the fitness value in this paper takes into account the global and local information of the image. The specific form is as follows:

$$fitness_{i}=\log(\log(sum(I_{ei})))*\frac{n\_edge(I_{ei})}{M_{i}\times N_{i}}*H(I_{i})-\log(\log(MSE(I_{i}))$$ (32)

Where *I*_*i*_ represents the image enhanced with the parameters of the corresponding position; *I*_*ei*_ is the sobel edge image of *I*_*i*_; *sum*(*I*_*ei*_) is the sum of the intensity values of all pixels of *I*_*ei*_; *n*_*edge*(*I*_*ei*_) is the number of such edge pixels with an intensity value greater than a certain fixed intensity value; *M*_*i*_×*N*_*i*_ is the size of *I*_*i*_, that is, the number of *I*_*i*_ pixels; *H*(*I*_*i*_) is the entropy value of image *I*_*i*_, the larger the value, the more information the image contains, its calculation method is as Formula (48); *MSE*(*I*_*i*_) is the image mean square error, the smaller the value indicates that the degree of image distortion is smaller, its calculation method is as Formula (33).


$$MSE=\frac{1}{M\times N}\sum_{i=1}^{M}\sum_{j=1}^{N}{\left|g(i,j)-h(i,j)\right|}^{2}$$ (33)

Where *g*(*i*,*j*) is the gray value of the pixels in row *i* and *j* column of the input image; *h*(*i*,*j*) is the gray value of the pixels in row *i* and column *j* of the enhanced image.

### 3.5 Image texture extraction

As shown in the framework flow of subsection 3.1, another direction is to extract the texture of the image. An image can usually be decomposed into a "structure-texture" form, it can be shown by Formula (34), reference [39, 40] extracts the main structure of the image by means of total variation regularization, namely Formula (35).


$$I=I_{S}+I_{T}$$ (34)

$$\underset{I_{S}}{\min}\sum_{x}{\left(I_{Sx}-I_{x}\right)}^{2}+\beta |\nabla (I_{Sx})|$$ (35)

Where *I* is the input, which can be the brightness channel; *x* is the image pixel index; *I*_*S*_ is the extracted structure image; data item (*I*_*Sx*_−*I*_*x*_)^2^ is to make the extracted structure similar to the structure in the input image; *β* is a weight value; $\sum_{x}\left|\nabla (I_{Sx})\right|$ is the total variational regularizer, written as:

$$\sum_{x}\left|\nabla (I_{Sx})\right|=\sum_{x}\left|\partial_{h}I_{Sx}\right|+|\partial_{v}I_{Sx}|$$ (36)

In the two-dimensional anisotropy expression, ∂_*h*_ and ∂_*v*_ are partial derivatives in two directions. Through experiments, it is found that this kind of total variation regularizer has limited ability in distinguishing strong structural edges and textures. For different images in the natural world, the texture characteristics are usually not fixed and without certain rules. In order to further highlight the texture and structural elements, a general pixel-level windowed total change metric *D*(*x*) and a new window inherent change metric *L*(*x*) are introduced here.to form a new regularizer, such as Formula (37):

$$\frac{D_{h}(x)}{L_{h}(x)+\varepsilon }+\frac{D_{v}(x)}{L_{v}(x)+\varepsilon }$$ (37)

$$D_{h}(x)=\sum_{y\in R(x)}g_{x,y}\cdot |{\left(\partial_{h}I_{S}\right)}_{y}|$$ (38)

$$D_{v}(x)=\sum_{y\in R(x)}g_{x,y}\cdot |{\left(\partial_{v}I_{S}\right)}_{y}|$$ (39)

$$L_{h}(x)=|\sum_{y\in R(x)}g_{x,y}\cdot {\left(\partial_{h}I_{S}\right)}_{y}|$$ (40)

$$L_{v}(x)=|\sum_{y\in R(x)}g_{x,y}\cdot {\left(\partial_{v}I_{S}\right)}_{y}|$$ (41)

Where *y*∈*R*(*x*), *R*(*x*) is rectangular areas centered on pixel *x*; *D*_*h*_(*x*) and *D*_*v*_(*x*) are the total window changes of pixel *x* in the directions of *h* and *v*; *L*_*h*_(*x*) and *L*_*v*_(*x*) are a new kind of inherent variation window. They are different from *D*_*h*_(*x*) and *D*_*v*_(*x*) in that they do not contain modulus, only the position of the absolute variation changes. The absolute variation from the gradient becomes the total absolute variation. Because the gradient variation has positive and negative values, the inherent variation of this window is almost zero in the undulating and uniform background area, and the variation is very large only at relatively large edges, and they pay more attention to the overall spatial change of the image; *ε* is a small positive number used to avoid the denominator being 0; *g*_*x*,*y*_ is a weighting function defined according to the spatial affinity, expressed as

$$g_{x,y}=\exp(-\frac{{\left(h_{x}-h_{y}\right)}^{2}+{\left(v_{x}-v_{y}\right)}^{2}}{2\sigma^{2}})$$ (42)

*g*_*x*,*y*_ is the gaussian kernel function, where *σ* is the space ratio for controlling window, which also affects the smoothness of the image to a certain extent. In summary, the new structure extraction method is as follows:

$$\underset{I_{S}}{\min}\sum_{x}{\left(I_{Sx}-I_{x}\right)}^{2}+\beta (\frac{D_{h}(x)}{L_{h}(x)+\varepsilon }+\frac{D_{v}(x)}{L_{v}(x)+\varepsilon })$$ (43)

**Fig 8:**
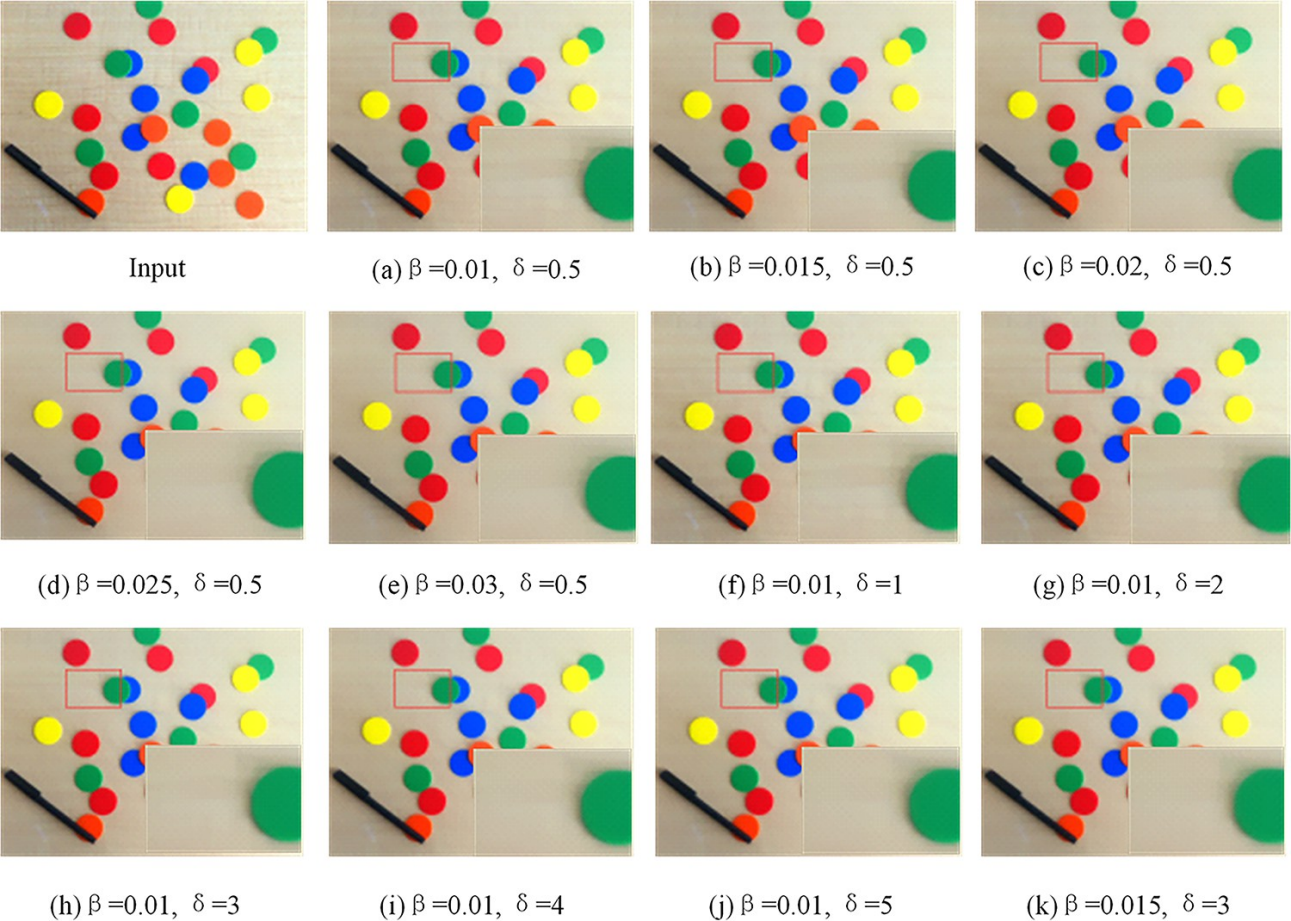
Parameter selection experiment.

The difference between Formula (43) and Formula (35) lies in the regularizer. The regularizer of Formula (43) is Formula (37), which adds a new kind of inherent variation window (40) and (41). The function of $\underset{I_{S}}{\min}\sum_{x}{\left(I_{Sx}-I_{x}\right)}^{2}$ is the same as before, *β* is a weight, it is also used to control the smoothness of the image. By adjusting the values of *β* and *σ*, the separation amount of image texture can be controlled to a certain extent. Generally, the value range of *β* is (0, 0.05), and the value range of *σ* is (0, 6). Through experiments, we find that when *β* slowly increases, the amount of texture separation of the image also increases, the texture separation is particularly obvious when *β* is set to 0.02; Similarly, when *σ* increases, the amount of texture separation will also increase, the separation of texture and structure is relatively clean when *σ* is set to 3. Considering the two parameters, and as shown in Table 1, we finally decide *β* = 0.015 and *σ* = 3. The effect is shown in Fig 8.

**Table 1:** Index values of images processed with different parameters.

	Input	a	b	c	d	e	f	g	h	i	j	k
PSNR	Inf	36.240	34.760	34.061	33.378	33.355	36.240	35.684	34.882	34.264	33.738	32.826
SSIM	1.000	0.977	0.972	0.970	0.968	0.967	0.977	0.975	0.971	0.969	0.968	0.967
Entropy	7.462	7.426	7.415	7.415	7.408	7.412	7.426	7.414	7.412	7.415	7.420	7.402

Finally, when the structure *I*_*S*_ is extracted, we can get the texture *I*_*T*_ of the image, namely:

$$I_{T}=I-I_{S}$$ (44)

### 3.6 Image texture addition

**Fig 9:**
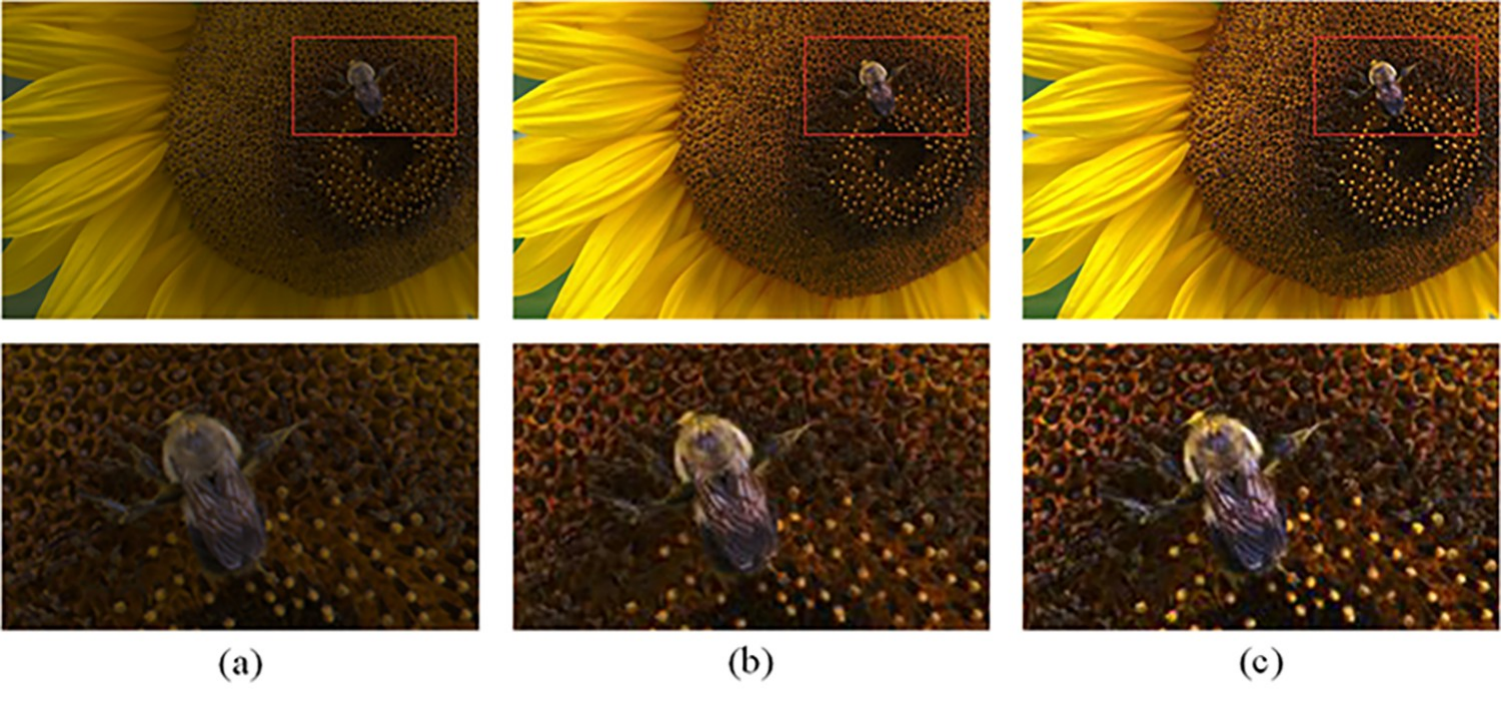
Image texture adding effect comparison. (a) Original image (b) Image processed by DHEDAPL (c) The image processed by DHEDAPL and adding detailed texture.

At the end of the enhancement framework process in subsection 3.1, we enhanced the image with DHEDAPL optimized based on improved CS to obtain an image *I*_*E*_ with suitable brightness and good contrast, but its disadvantage is that the image details are not obvious enough. While we strip out the detail information *I*_*T*_ in the original image through the new total variational regularization method described in subsection 3.5. At this time, we can add *I*_*T*_ to *I*_*E*_, which can enrich the details of the image, and this operation has little effect on the brightness characteristics and contrast characteristics of *I*_*E*_ at the same time. As shown in Fig 9, the effects of performing separate DHEDAPL processing on an image and adding texture details of the original image after DHEDAPL processing are compared, it can be seen that after the original image is processed by DHEDAPL, the brightness and contrast are improved significantly, but the image details are smoother. On this basis, the original image texture details are added, the overall visual effect of the image is good, and the brightness contrast is suitable for human eyes. View the enlarged details of the image, the image with texture details *I*_*T*_ contains more details and the texture is clearer. Therefore, the final output image of the method proposed in this paper is represented by the following definition:

$$I_{O}=I_{E}+I_{T}$$ (45)

Where *I*_*O*_ is the final output image we obtained, *I*_*E*_ is the image obtained after the original image is processed by DHEDAPL, and *I*_*T*_ is the texture details of the original image.

## 4 Experiments

In this section, we shown the experimental results of the proposed method for low-light image. All the codes in this method are run in matlabR2019a, and all the experiments are performed on a PC with operating system windows10, 4GB memory and 3.20GHz i5 CPU. The test images selected in this article are all low-light images, which can be divided into three categories. Due to the limited space of the article, the following are selected as the representatives for display. The test images selected in this article are all low-light images, which can be divided into three groups: (1) 20 random images from MIT-Adobe 5K data set [41]; (2) The first 100 images from MIT-Adobe 5K data set; (3) 64 images from DICM data set [42]. The first two groups of images are paired images, and the third group of images are unpaired images. These images all have the characteristics of low brightness, unclear image details and low contrast. In this chapter, we first verify the anti-noise performance of the new total variational model in making image texture mask, and compare it with the unsharp mask method, which is the classical method of making mask [43]. In addition, we have compared the proposed method with other mainstream algorithms. The contrast algorithm includes the adaptive gamma algorithm AGCWD in [44], Al-Ameen proposed the new image illumination enhancement method [45], dual-platform limit-based double histogram equalization (BHE2PL) [33], the biologically inspired multi-exposure fusion framework method (BIMEF) [46], brightness-maintained dynamic histogram equalization (BPDHE) [11], a simple yet effective low-light image enhancement (LIME) method [22], multi-scale retinex with color restoration algorithm (MSRCR) [21], the nature preservation enhancement algorithm (NPEA) in [47] and the simultaneous reflectance and illumination estimation method (SRIE) in [48]. Therefore, there are three sections in this chapter: (1) Anti-noise performance analysis of texture mask made by the new total variational model; (2) Subjective evaluation of the experimental results; (3) Objective evaluation of the experimental results of the proposed method.

### 4.1 Anti-noise performance analysis

**Fig 10:**
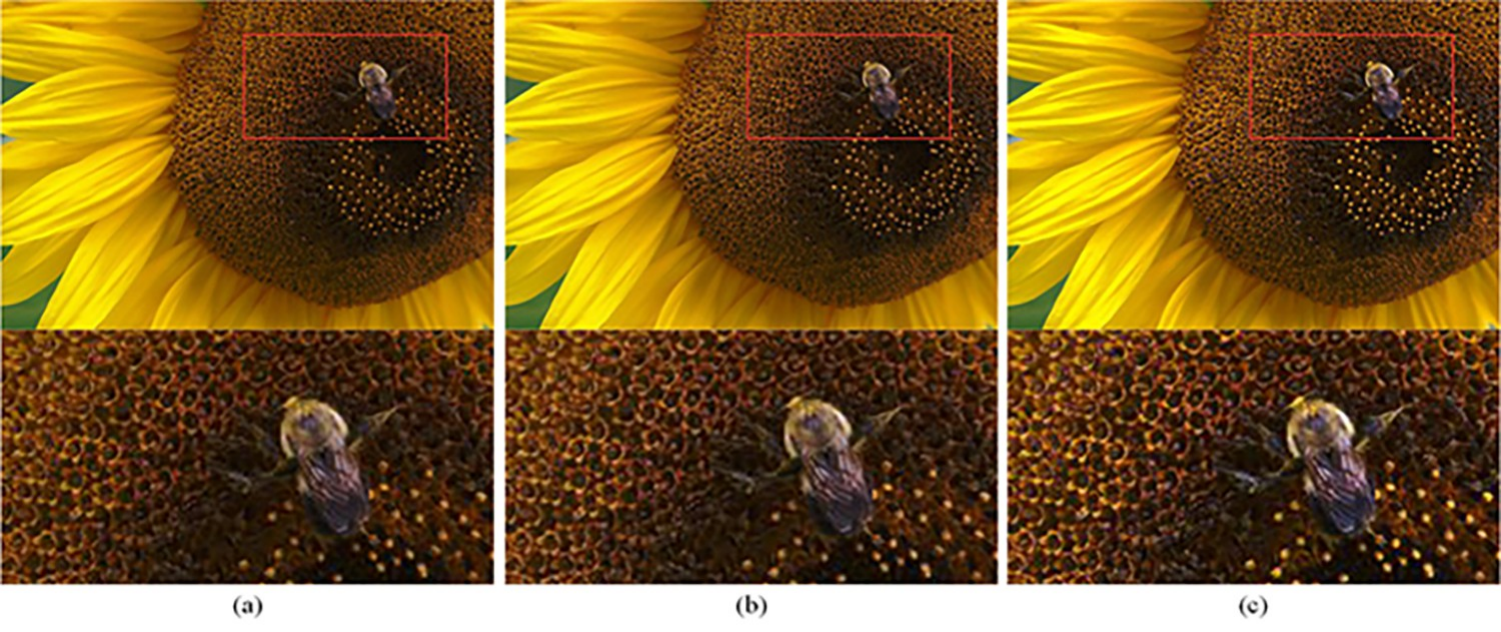
Image texture adding effect comparison. (a) Image processed by DHEDAPL (b) Image processed by DHEDAPL adding unsharp mask (c) Image processed by DHEDAPL adding texture mask.

In chapter 3, we have introduced the method in detail, in order to enrich the texture details of the image processed by DHEDAPL, we try to use a new total variational model to extract the image texture information and avoid noise as much as possible. In this section, we verify it through experiments, and analyze the image information contained in the image before and after adding texture information. At the same time, we also compare it with the unsharp mask, which is the classic method [43]. As shown in the Fig 10, we show the changes of the two images before and after adding texture information. Although the images processed by DHEDAPL have been satisfactorily evaluated by human eyes in terms of contrast and brightness, the image details are not clear enough and there are few image details; The contrast of the image with unsharp mask is improved, but not too much image detail is added; After the texture details extracted by the new total variational model are added to the DHEDAPL processed image, we can obviously feel the richness of details, such as several details shown in the figure, there are bees and stamens.

In addition, we conducted comparative experiments on 20 randomly selected images in MIT-5K data set. From the data results shown in the Table 1, there are the average values of the four evaluation values of 20 images before and after adding texture information, which are peak signal to noise ratio (PSNR), structural similarity (SSIM), average gradient (AG) and natural image quality evaluator (NIQE) respectively. PSNR and SSIM are full reference metrics. PSNR is the ratio of the energy of the image peak signal to the average energy of noise, which can be used to evaluate the image noise content, the larger the value, the less the image noise and the better the image quality; SSIM is an index to measure the similarity between two images, its value range is (0,1), the closer the value is to 1, the more complete the image structure is and the better the image quality is; AG and NIQE are non-reference metrics, in which the value of AG reflects the gradient detail contained in the image, the larger the value, the more image detail information; The blind image quality evaluation index NIQE is a relatively comprehensive image evaluation index, the smaller its value, the better the image visual feeling. It can be seen from the data in the Table 2, the AG value of the image with unsharp mask and the image with texture mask has been improved compared with the previous images, especially the image with texture mask; But the probability of noise increased by adding unsharp mask is very high. From the average value of PSNR of 20 images, it can be seen that the PSNR value of the image added with unsharp mask decreases, but the PSNR of the image does not decrease after adding the texture information extracted by the new total variational model, even the PSNR value is improved; For the SSIM index, there also has the same evaluation. Moreover, for the NIQE index, the image with texture information extracted by the new total variational model has also received the best evaluation. Therefore, it is enough to show that our model not only increases the image texture information, but also does not introduce too much noise to destroy the image quality and structure, and achieves the purpose of enriching the image details.

**Table 2:** Average value of four evaluation indexes of 20 random images of MIT-5K.

	DHEDAPL	Unsharp mask	Ours
**PSNR**	22.2326	21.6499	**22.4175**
**SSIM**	0.8301	0.8181	**0.8313**
**AG**	6.3646	6.7872	**7.2978**
**NIQE**	2.9636	2.9837	**2.8562**

### 4.2 Subjective evaluation

The visual perception of an image is one aspect of evaluating image quality, and people’s perception of an image includes various aspects such as color, brightness, and clarity. Different algorithms have different degrees of improvement in various aspects of low-light images, as shown in Figs 11–30, which are the effect of each test image processed by various methods, from these experimental results, our method as a whole has the greatest improvement in image visual effects compared to other algorithms.

**Fig 11:**
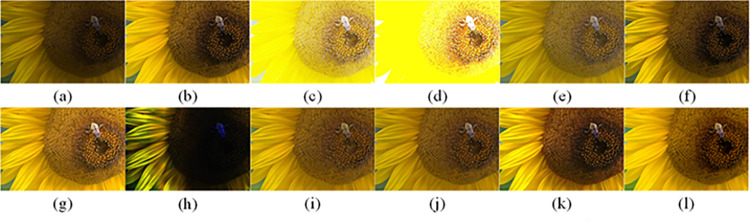
Comparisons of the ten algorithms in enhancing low-light image 1. (a) low-light image (b) AGCWD (c) Al-Ameen (d) BHE2PL (e) BIMEF (f) BPDHE (g) LIME (h) MSRCR (i) NPEA (j) SRIE (k) OURS (l) Normal light image.

**Fig 12:**
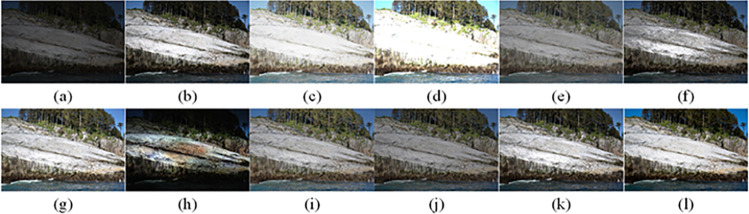
Comparisons of the ten algorithms in enhancing low-light image 2. (a) low-light image (b) AGCWD (c) Al-Ameen (d) BHE2PL (e) BIMEF (f) BPDHE (g) LIME (h) MSRCR (i) NPEA (j) SRIE (k) OURS (l) Normal light image.

**Fig 13:**
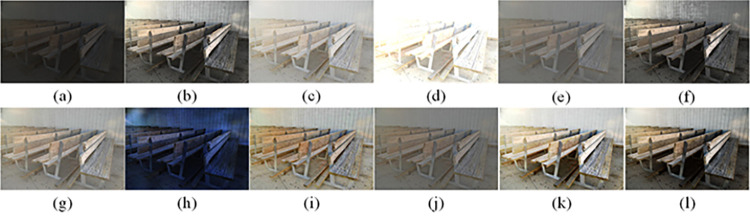
Comparisons of the ten algorithms in enhancing low-light image 3. (a) low-light image (b) AGCWD (c) Al-Ameen (d) BHE2PL (e) BIMEF (f) BPDHE (g) LIME (h) MSRCR (i) NPEA (j) SRIE (k) OURS (l) Normal light image.

**Fig 14:**
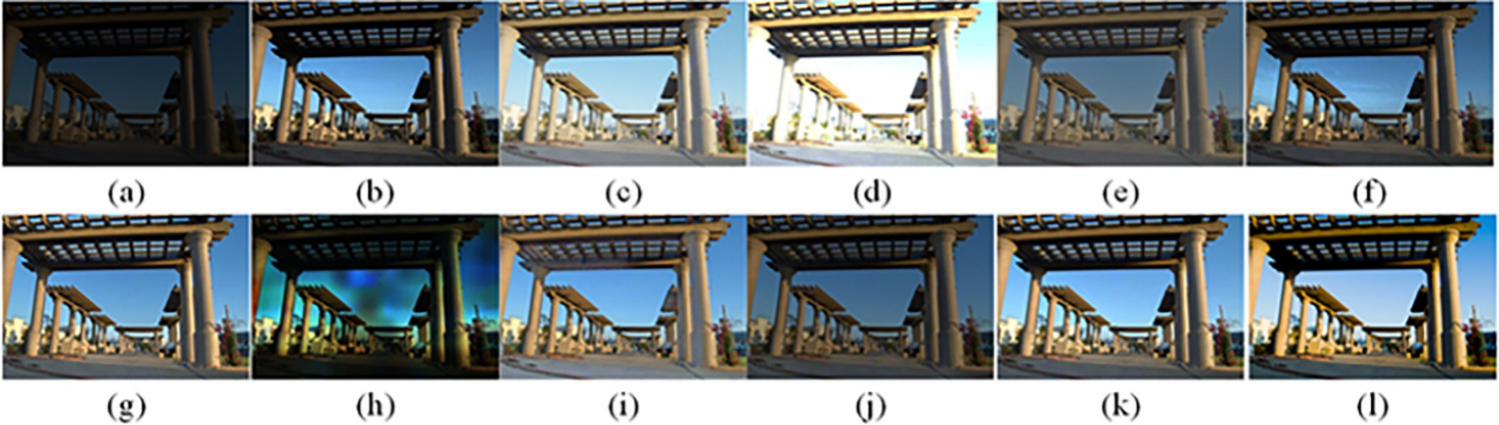
Comparisons of the ten algorithms in enhancing low-light image 4. (a) low-light image (b) AGCWD (c) Al-Ameen (d) BHE2PL (e) BIMEF (f) BPDHE (g) LIME (h) MSRCR (i) NPEA (j) SRIE (k) OURS (l) Normal light image.

**Fig 15:**
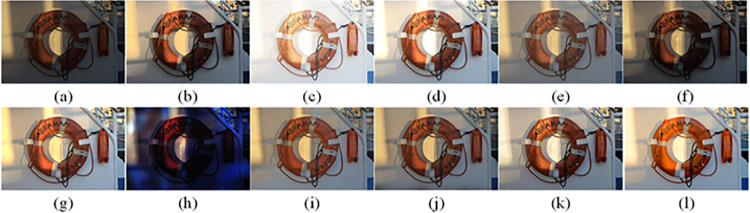
Comparisons of the ten algorithms in enhancing low-light image 5. (a) low-light image (b) AGCWD (c) Al-Ameen (d) BHE2PL (e) BIMEF (f) BPDHE (g) LIME (h) MSRCR (i) NPEA (j) SRIE (k) OURS (l) Normal light image.

**Fig 16:**
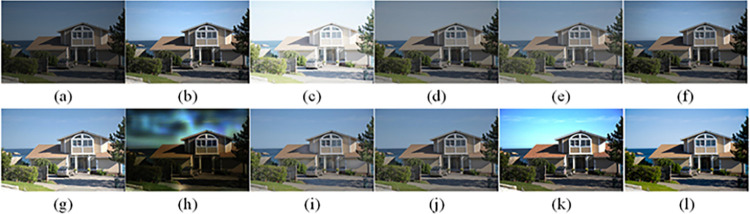
Comparisons of the ten algorithms in enhancing low-light image 6. (a) low-light image (b) AGCWD (c) Al-Ameen (d) BHE2PL (e) BIMEF (f) BPDHE (g) LIME (h) MSRCR (i) NPEA (j) SRIE (k) OURS (l) Normal light image.

**Fig 17:**
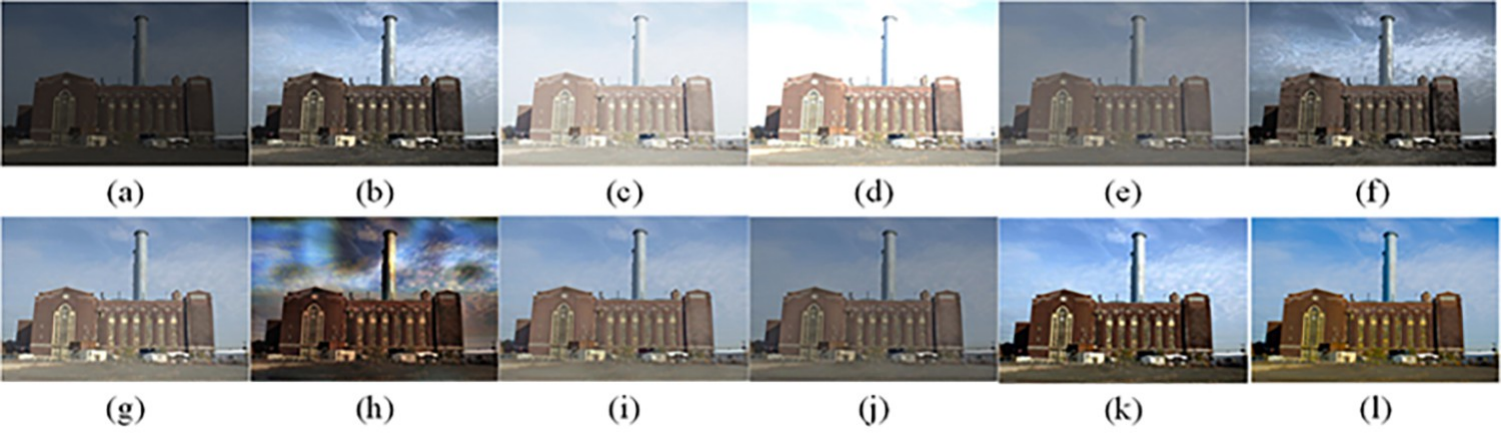
Comparisons of the ten algorithms in enhancing low-light image 7. (a) low-light image (b) AGCWD (c) Al-Ameen (d) BHE2PL (e) BIMEF (f) BPDHE (g) LIME (h) MSRCR (i) NPEA (j) SRIE (k) OURS (l) Normal light image.

**Fig 18:**
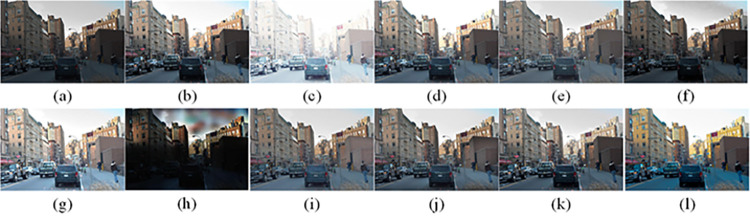
Comparisons of the ten algorithms in enhancing low-light image 8. (a) low-light image (b) AGCWD (c) Al-Ameen (d) BHE2PL (e) BIMEF (f) BPDHE (g) LIME (h) MSRCR (i) NPEA (j) SRIE (k) OURS (l) Normal light image.

**Fig 19:**
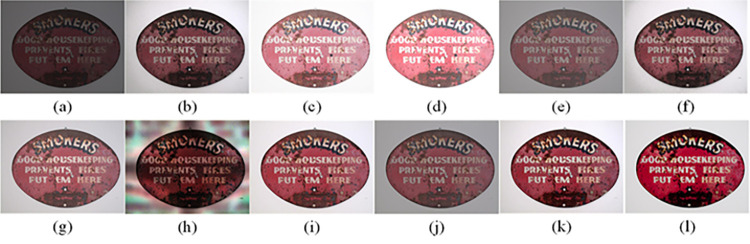
Comparisons of the ten algorithms in enhancing low-light image 9. (a) low-light image (b) AGCWD (c) Al-Ameen (d) BHE2PL (e) BIMEF (f) BPDHE (g) LIME (h) MSRCR (i) NPEA (j) SRIE (k) OURS (l) Normal light image.

**Fig 20:**
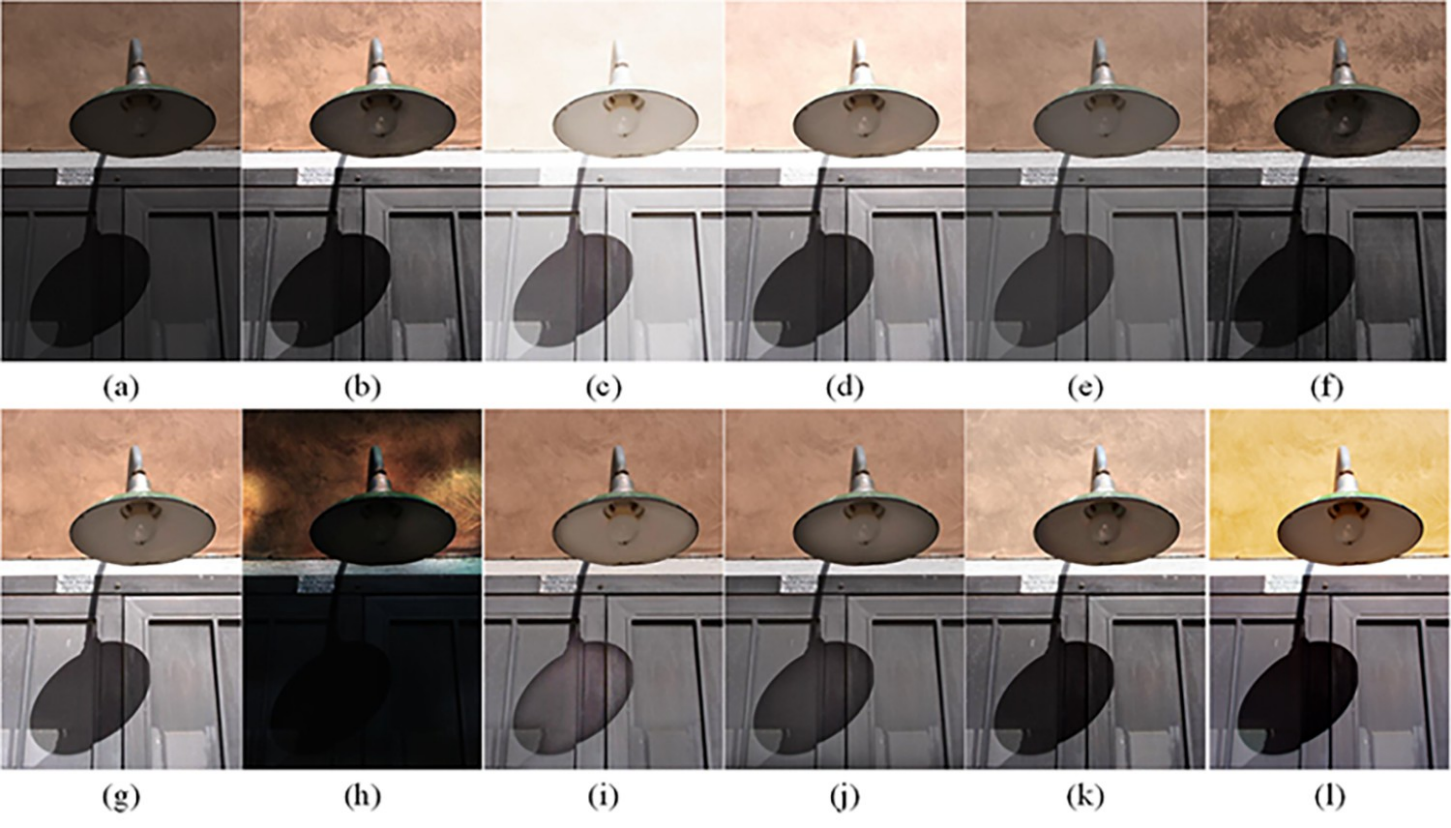
Comparisons of the ten algorithms in enhancing low-light image 10. (a) low-light image (b) AGCWD (c) Al-Ameen (d) BHE2PL (e) BIMEF (f) BPDHE (g) LIME (h) MSRCR (i) NPEA (j) SRIE (k) OURS (l) Normal light image.

**Fig 21:**
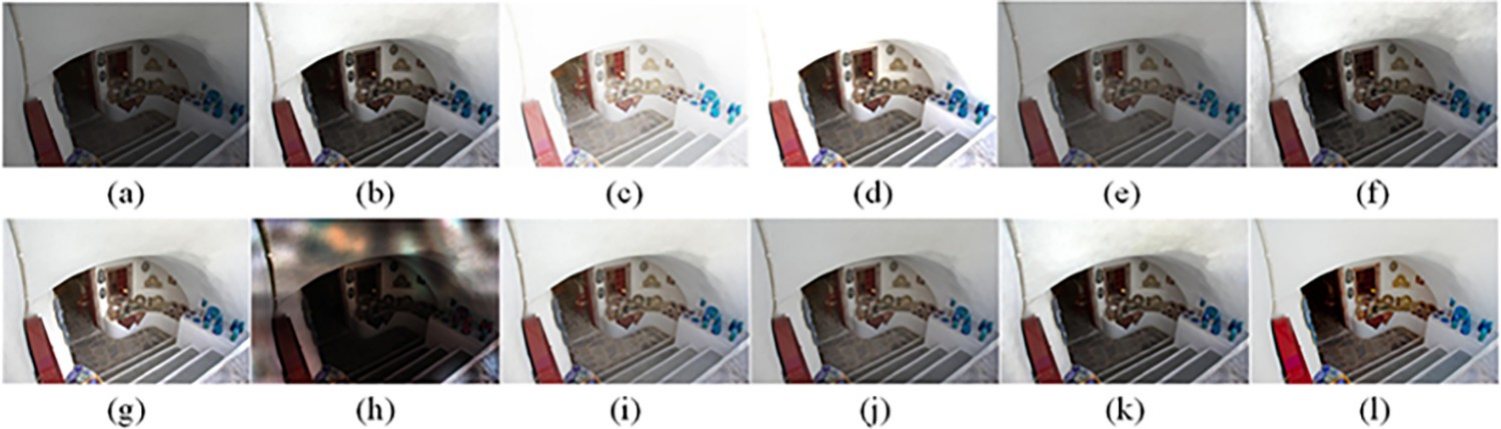
Comparisons of the ten algorithms in enhancing low-light image 11. (a) low-light image (b) AGCWD (c) Al-Ameen (d) BHE2PL (e) BIMEF (f) BPDHE (g) LIME (h) MSRCR (i) NPEA (j) SRIE (k) OURS (l) Normal light image.

**Fig 22:**
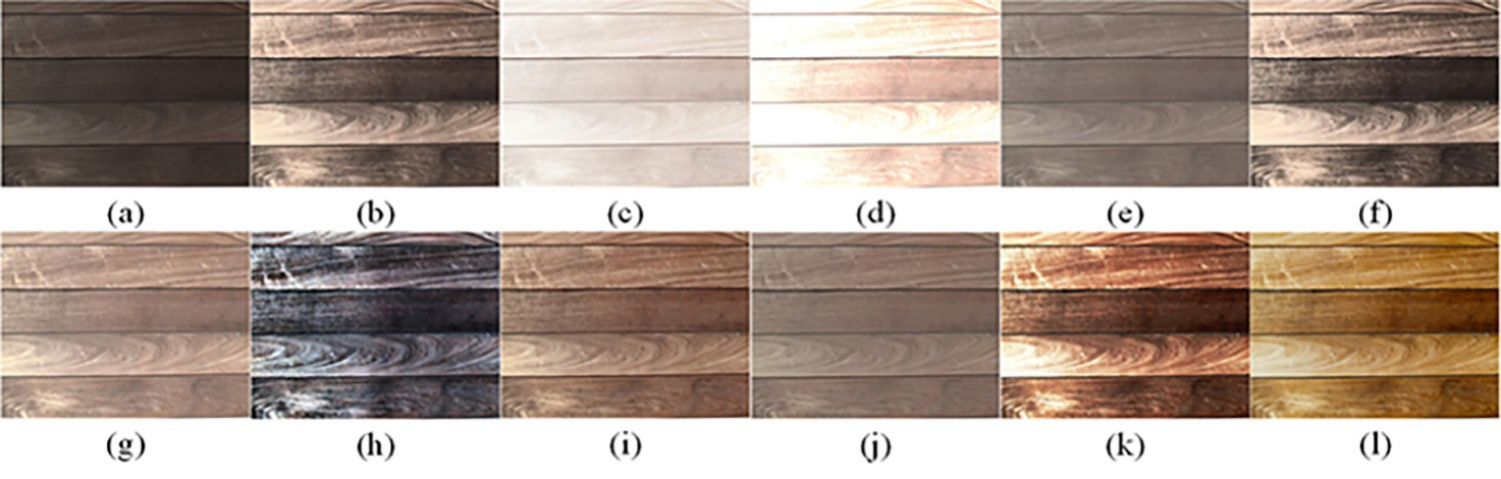
Comparisons of the ten algorithms in enhancing low-light image 12. (a) low-light image (b) AGCWD (c) Al-Ameen (d) BHE2PL (e) BIMEF (f) BPDHE (g) LIME (h) MSRCR (i) NPEA (j) SRIE (k) OURS (l) Normal light image.

**Fig 23:**
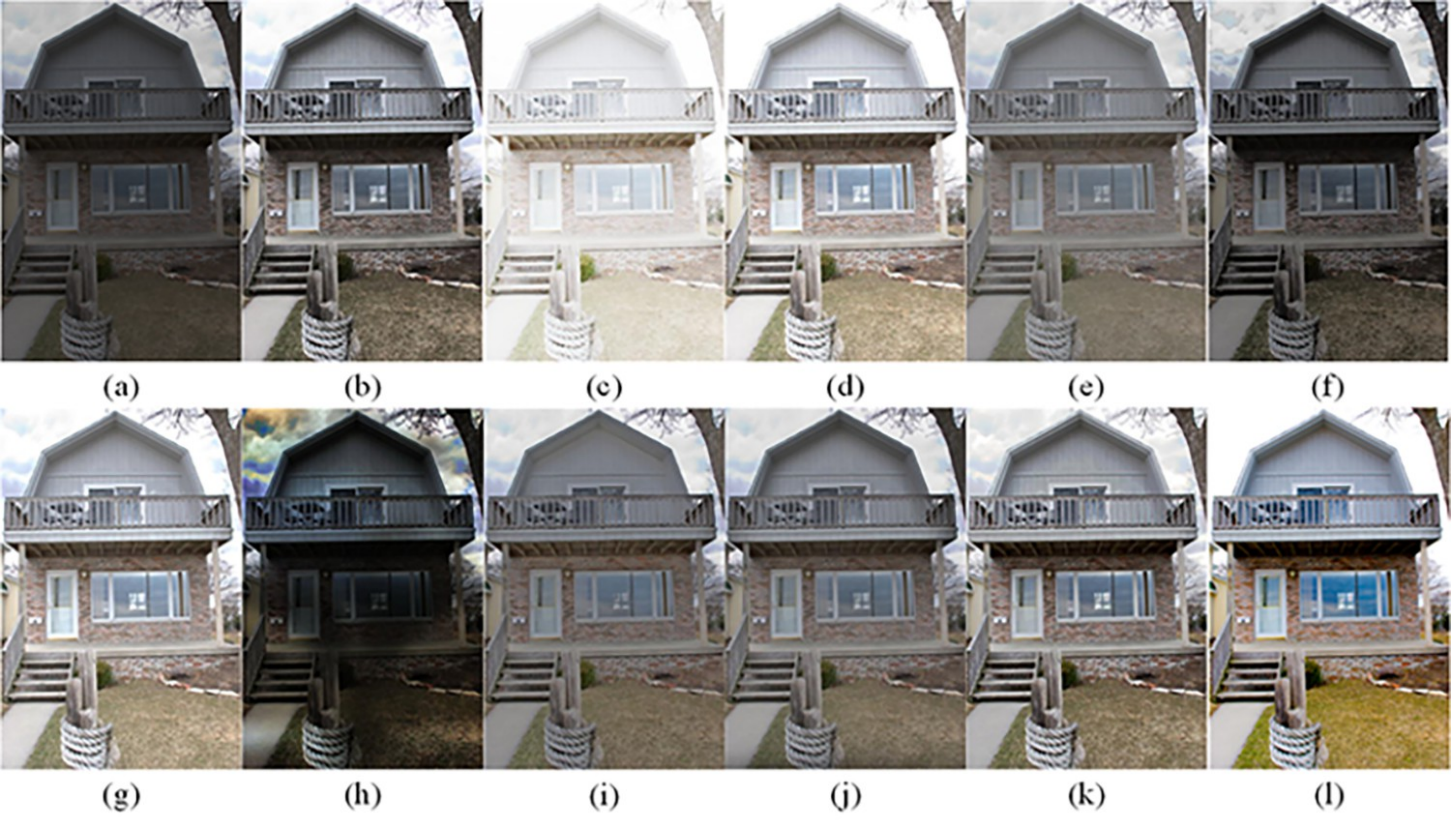
Comparisons of the ten algorithms in enhancing low-light image 13. (a) low-light image (b) AGCWD (c) Al-Ameen (d) BHE2PL (e) BIMEF (f) BPDHE (g) LIME (h) MSRCR (i) NPEA (j) SRIE (k) OURS (l) Normal light image.

**Fig 24:**
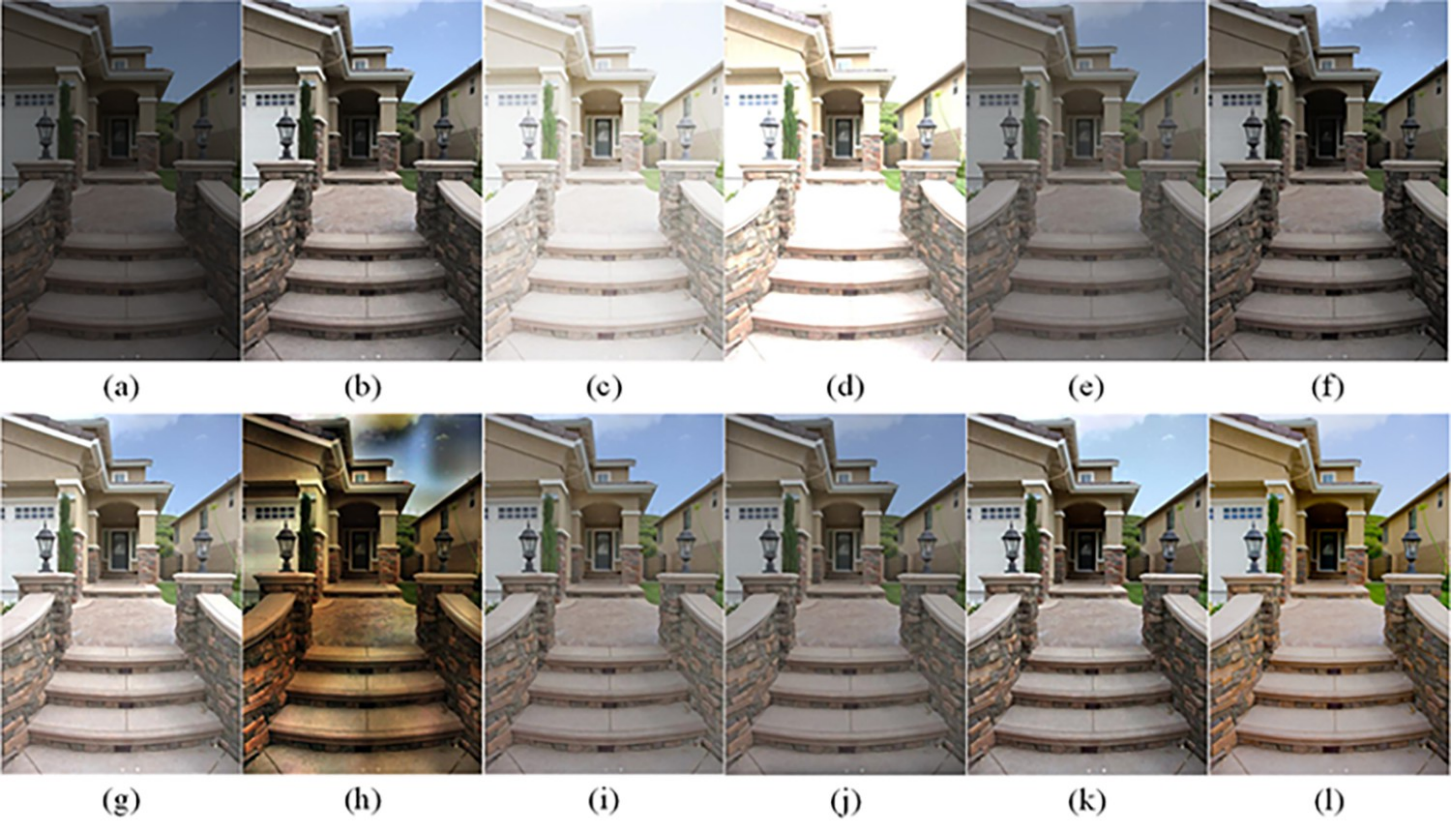
Comparisons of the ten algorithms in enhancing low-light image 14. (a) low-light image (b) AGCWD (c) Al-Ameen (d) BHE2PL (e) BIMEF (f) BPDHE (g) LIME (h) MSRCR (i) NPEA (j) SRIE (k) OURS (l) Normal light image.

**Fig 25:**
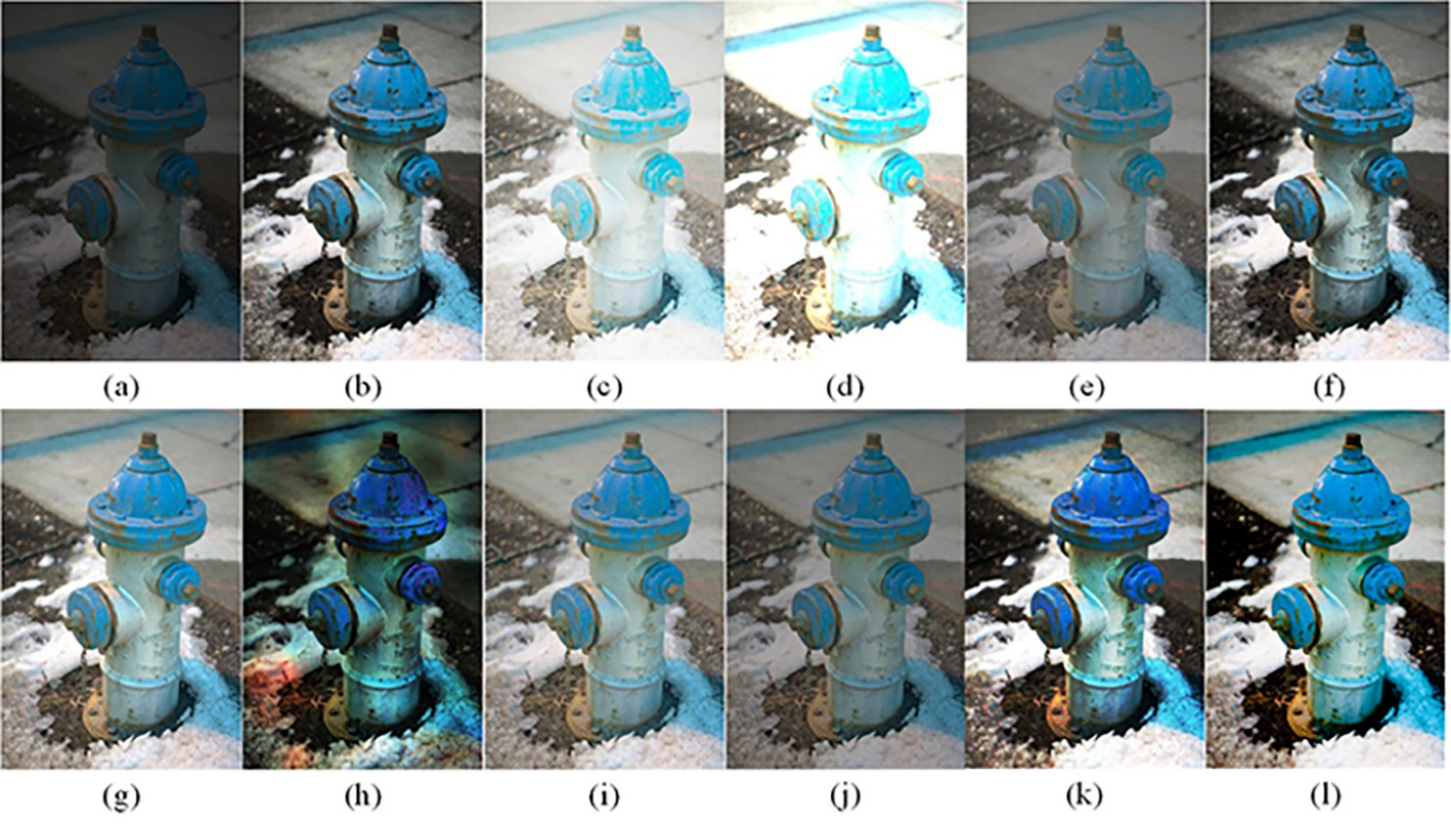
Comparisons of the ten algorithms in enhancing low-light image 15. (a) low-light image (b) AGCWD (c) Al-Ameen (d) BHE2PL (e) BIMEF (f) BPDHE (g) LIME (h) MSRCR (i) NPEA (j) SRIE (k) OURS (l) Normal light image.

**Fig 26:**
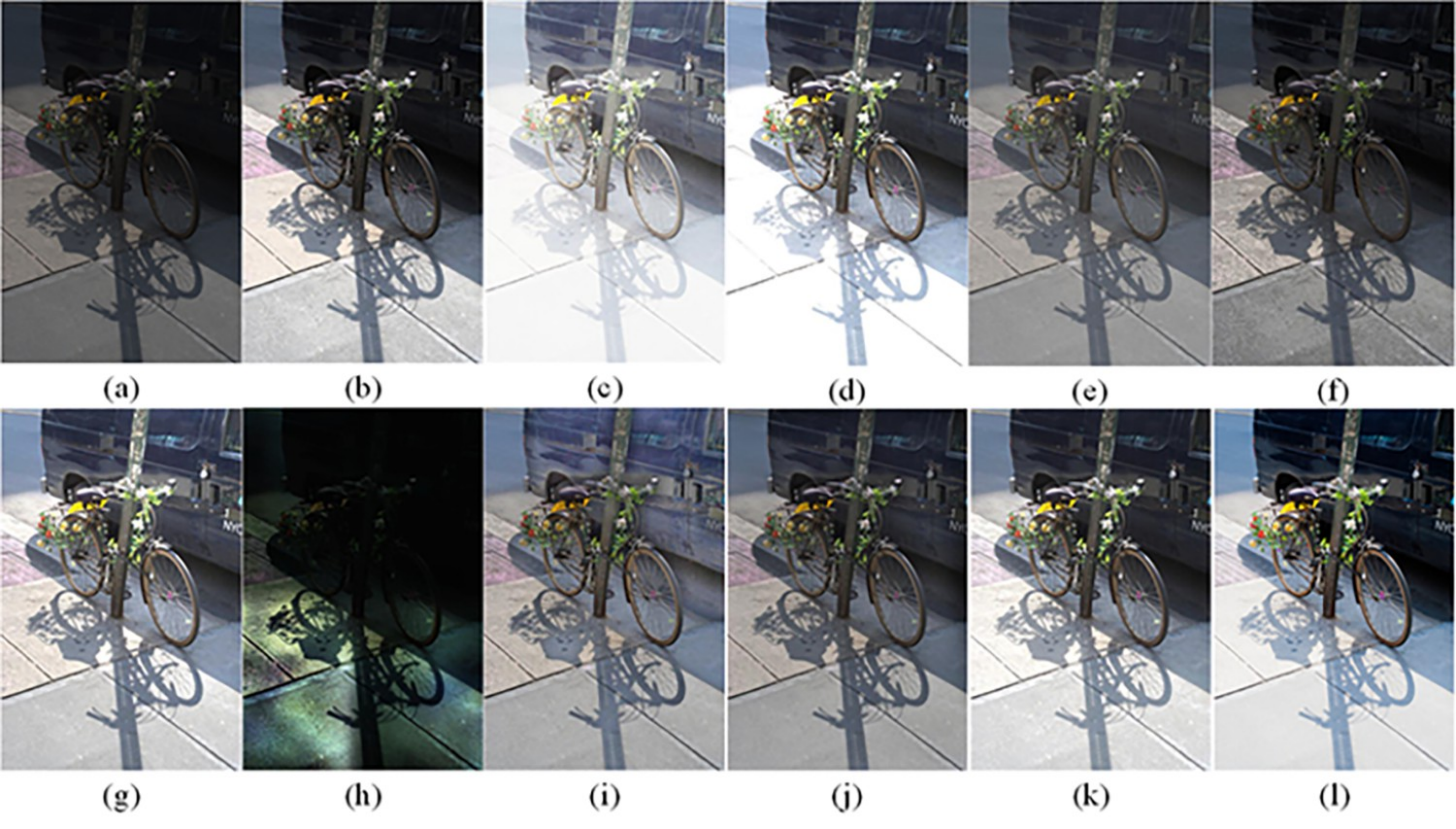
Comparisons of the ten algorithms in enhancing low-light image 16. (a) low-light image (b) AGCWD (c) Al-Ameen (d) BHE2PL (e) BIMEF (f) BPDHE (g) LIME (h) MSRCR (i) NPEA (j) SRIE (k) OURS (l) Normal light image.

**Fig 27:**
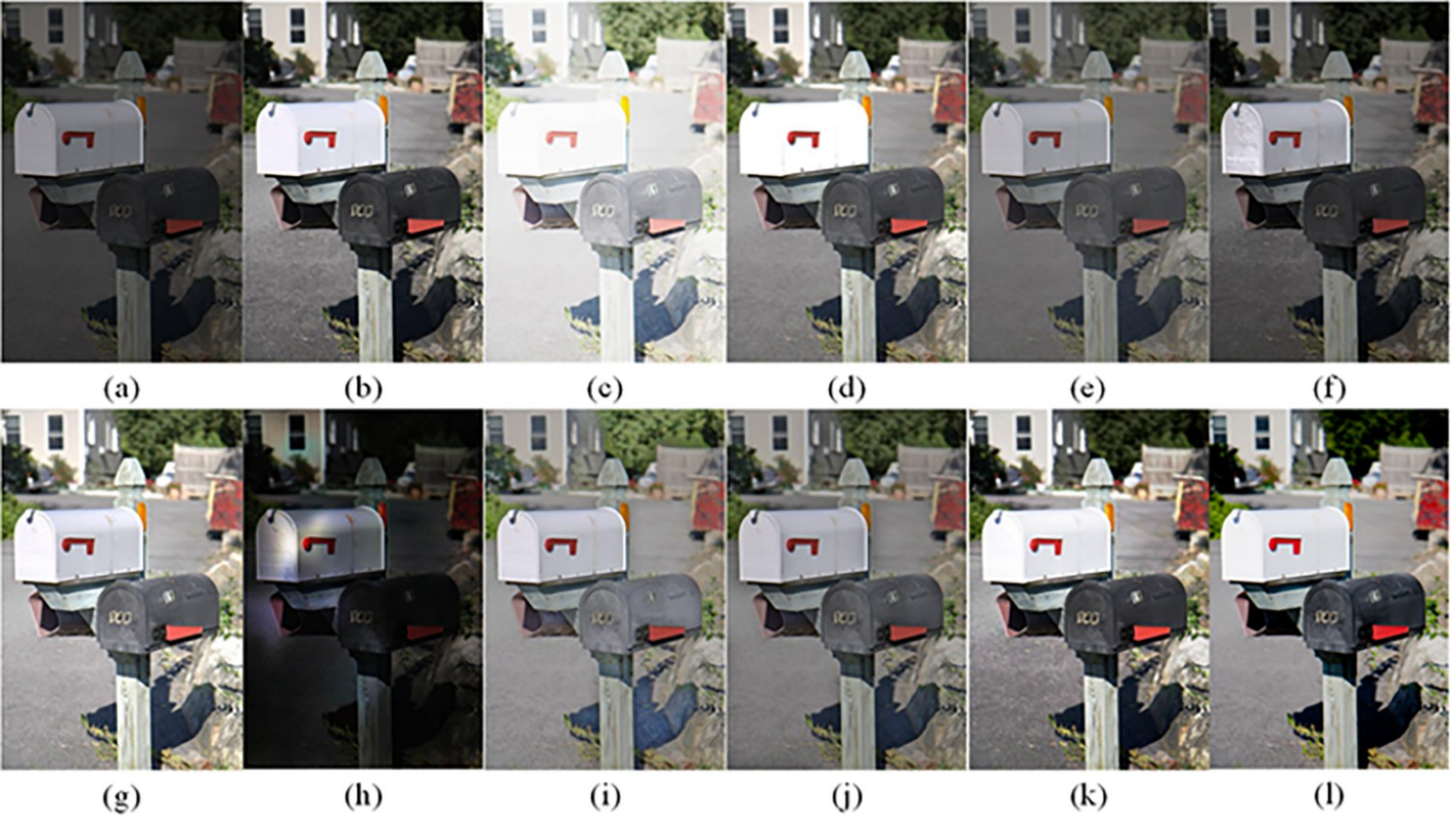
Comparisons of the ten algorithms in enhancing low-light image 17. (a) low-light image (b) AGCWD (c) Al-Ameen (d) BHE2PL (e) BIMEF (f) BPDHE (g) LIME (h) MSRCR (i) NPEA (j) SRIE (k) OURS (l) Normal light image.

**Fig 28:**
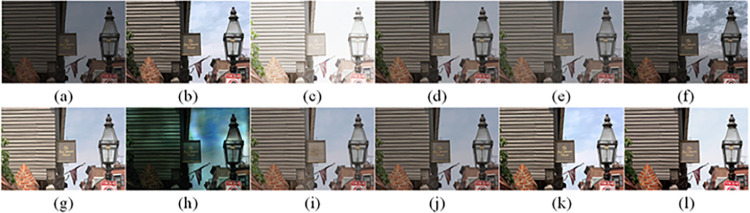
Comparisons of the ten algorithms in enhancing low-light image 18. (a) low-light image (b) AGCWD (c) Al-Ameen (d) BHE2PL (e) BIMEF (f) BPDHE (g) LIME (h) MSRCR (i) NPEA (j) SRIE (k) OURS (l) Normal light image.

**Fig 29:**
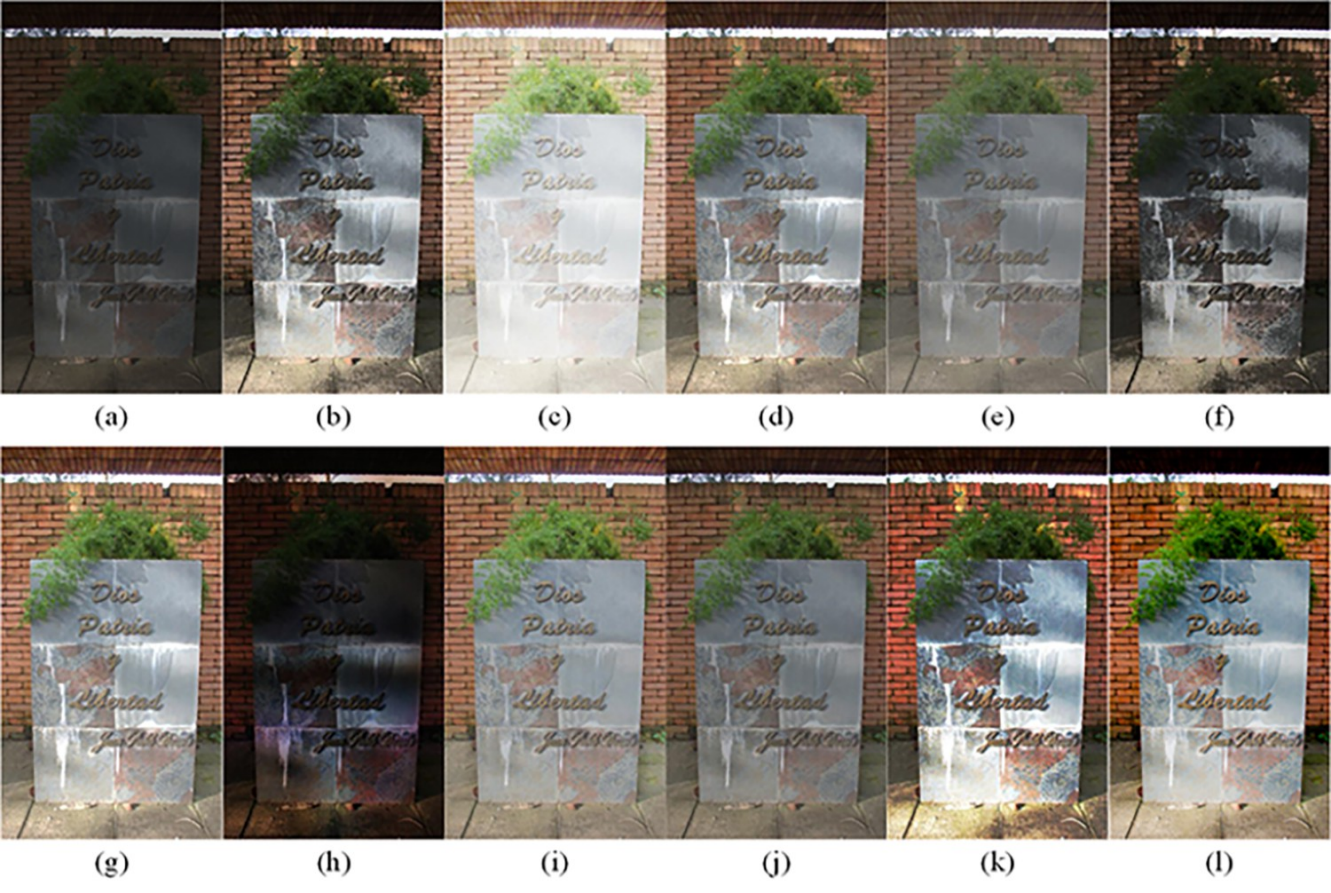
Comparisons of the ten algorithms in enhancing low-light image 19. (a) low-light image (b) AGCWD (c) Al-Ameen (d) BHE2PL (e) BIMEF (f) BPDHE (g) LIME (h) MSRCR (i) NPEA (j) SRIE (k) OURS (l) Normal light image.

**Fig 30:**
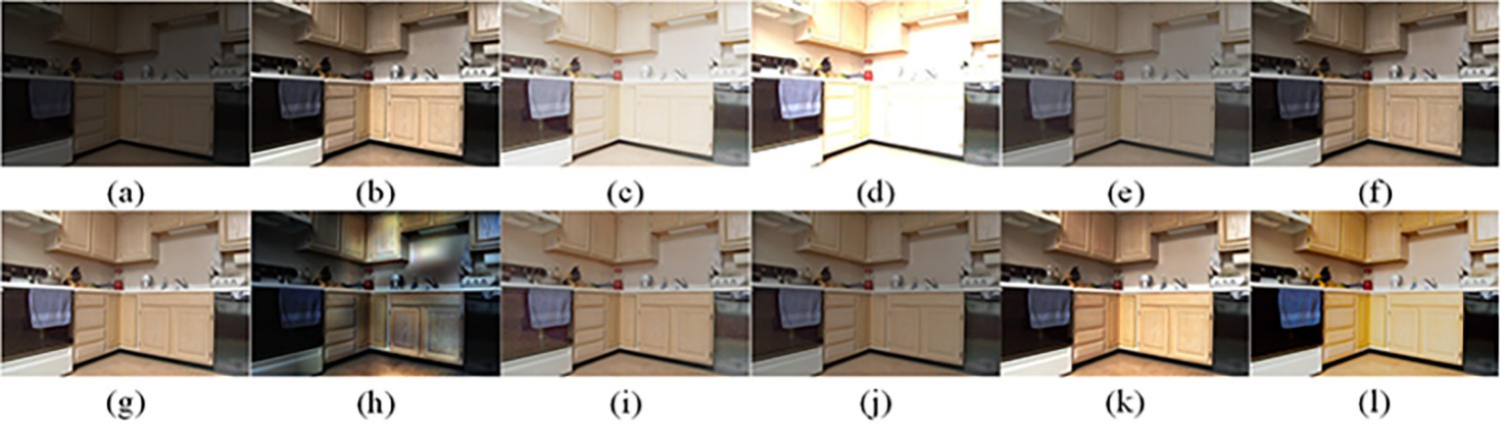
Comparisons of the ten algorithms in enhancing low-light image 20. (a) low-light image (b) AGCWD (c) Al-Ameen (d) BHE2PL (e) BIMEF (f) BPDHE (g) LIME (h) MSRCR (i) NPEA (j) SRIE (k) OURS (l) Normal light image.

AGCWD is one of the derivative methods of gamma correction based on adjusting the brightness of the image. It is outstanding when adjusting the brightness of the general image. However, in this article, AGCWD shows an unstable effect when processing low-light images. For some images, it can get good results, but for some images, there are still unsatisfactory results. In addition, the brightness of the overall output image is dark, and the image contains dark areas obviously, such as trees and rivers in Fig 11(B), the ground in Figs 13(B) and 14(B), lamp in Fig 20(B), cars in Fig 26(B), etc.

Al-Ameen’s method is also aimed at improving the brightness of the image, but it can be clearly seen in the experimental part of this article, the Al-Ameen’ method has an excessive enhancement effect on low-light images, such as Figs 11(C), 15(C), 16(C), 18(C), 20(C) and 23(C). On the whole, the output images are whitened, and the image details are too smooth.

The effect of BHE2PL is similar to that of Al-Ameen’s method, with over enhanced effect and even serious exposure, such as Figs 11(D)–14(D), 17(D), 18(D), 21(D), 24(D) and 30(D), especially for images Figs 14(D), 17(D), 18(D) and 24(D) with sky area.

BIMEF is a fusion-based approach. The structure of the image enhanced by BIMEF is well preserved, the image brightness has been improved to a certain extent, the image information content has increased, but the contrast is not obvious. From the image experimental results, the image processed by BIMEF is always dim in color, and even has a hazy feeling.

BPDHE is relatively stable in terms of color output, but when processing the image, the brightness of the image is unevenly improved, and many dark areas in the image are not well corrected, such as the lifebuoy in Fig 15(F), the lawn in Fig 17(F), the car in Fig 18(F), the light bulb in Fig 20(F), the car in Fig 26(F), the trees in Fig 27(F), etc., so that these subjects in the image are not clear. Secondly, the color of the sky area in some images is gray, such as Figs 17(F), 18(F) and 24(F), and the clouds in the sky in Figs 23(F) and 28(F) are strange.

The LIME algorithm is based on retinex theory, it mainly tends to estimate the illumination map of low illumination image to realize enhancement. This algorithm has stable enhancement effect and is often used as a comparative experiment of image enhancement algorithm. In our experimental results, it also shows a good processing effect. The overall visual feeling of the image is comfortable, but the texture details of individual images are not rich enough, such as the wall and wooden chair in Fig 13(G), the building in Fig 17(G), the text in Fig 19(G), the wood board in Fig 22(G) and the fire hydrant in Fig 25(G). In addition, the colors of Figs 13(G), 17(G), 19(G), 23(G), 29(G) and 30(G) are slightly dim.

MSRCR is a classical multi-scale retinex algorithm. From the experimental results, there are serious artifacts in its processing of low illumination images is the same as other images.

NPEA has achieved good results in nature protection. This method is relatively good in color stability and overall structure maintenance, but the image contrast is not obvious after processing. In addition, the restoration of the light and shadow of the image is very unnatural and does not conform to the normal natural phenomenon, as shown in Figs 20(I) and 27(I), here is an obvious bubble feeling at the light and shadow.

Most of the images processed by the SRIE method are close to those processed by NPEA, and the color of the processed image is relatively stable, but the overall brightness of the output image is darker than that of NPEA, such as Figs 12(J), 13(J), 14(J), 17(J), 19(J), 22(J), 29(J), etc. Because of the brightness problem, these images have a hazy feeling as a whole, and the visual effect is not very good, Compared with normal light images, there is still a certain gap.

The method proposed in this paper has comprehensive performance in this experiment. By observing the effects of each method in each image, the method in this paper shows the effect most suitable for human observation. Compared with other methods, the method proposed in this paper is also closest to the image taken under normal light. In Fig 11, the image content includes petals, stamens and bees. It is difficult for other methods to enhance image contrast, maintain image color and restore image details at the same time. Our method has completed these tasks well. Through observation, the small details in the image have been well maintained, one is stamens and the other is bees. Moreover, in the stamen part, we can not only restore the details, but also maintain the sense of color hierarchy, and the fine hairs of bees can be seen clearly. For other images, the effect is also excellent. The overall image contrast and brightness are suitable for human eye observation, with natural color and rich details. From these experimental results, compared with other algorithms, our method has the best evaluation in image visual effect.

### 4.3 Objective index evaluation

In order to evaluate the quality of experimental images more objectively, this paper uses several metrics such as image peak signal-to-noise ratio (PSNR), image structural similarity (SSIM), image information entropy (Entropy), image average gradient (AG), image contrast ratio (CR), image brightness difference (BD), image standard deviation (STD) and natural image quality evaluator (NIQE) to evaluate the experimental images from different angles. Next, we describe these indicators in detail.

#### 4.3.1 Image peak signal-to-noise ratio (PSNR)

PSNR is the most extensive and commonly used objective evaluation method to measure the effect of image denoising. The larger the PSNR value, the less the image noise content and the better the image quality. The specific calculation method of PSNR is shown in Formula (46).


$$PSNR=10\log_{10}\frac{MaxValue^{2}}{MSE}=10\log_{10}\frac{{\left(2^{bits}-1\right)}^{2}}{MSE}$$ (46)

Where, *MaxValue* is the maximum pixel value of the image, in this process, the gray value of RGB three channels shall be processed; *bits* is generally taken as 8; The *MSE* measure represents the direct deviation between the enhanced image and the reference image, its calculation method has been introduced in Formula (33).

From the value of PSNR in Table 3, our method performs best in the experiment of 20 random images of MIT-5K, followed by AGCWD and LIME. In our method, the PSNR value of each image can be ranked in the top three, and most of them are the first. And as show in Table 4, in the experiment of the top 100 images of MIT-5K, the performance of the method in this paper is not weak, ranking second only to AGCWD. It shows that our method has a good effect on improving image quality.

**Table 3:** PSNR values of 20 random images of MIT-5K.

PSNR	AGCWD	Al-Ameen	BHE2PL	BIMEF	BPDHE	LIME	MSRCR	NPEA	SRIE	OURS
**pic1**	**24.905**	10.037	7.809	17.732	18.970	18.421	10.955	**21.436**	19.252	**22.597**
**pic2**	**24.193**	11.709	9.372	15.501	17.946	**18.603**	11.952	17.531	15.103	**24.917**
**pic3**	**25.382**	9.881	4.887	14.052	**20.439**	13.101	10.996	12.868	14.492	**24.973**
**pic4**	**16.925**	14.320	8.749	13.277	14.907	**17.757**	10.829	15.740	12.167	**20.723**
**pic5**	15.500	11.666	**20.299**	16.166	10.019	**22.389**	7.388	16.514	15.239	**20.386**
**pic6**	**24.170**	8.987	15.856	15.810	17.157	15.728	10.132	**17.282**	17.007	**20.399**
**pic7**	**20.939**	9.320	8.474	16.201	18.376	17.080	14.121	**19.111**	17.197	**21.153**
**pic8**	**23.146**	7.962	**21.591**	20.053	16.358	14.465	9.633	18.615	20.233	**22.005**
**pic9**	18.221	11.243	10.413	12.630	**18.900**	16.191	11.803	**19.167**	13.865	**21.994**
**pic10**	**19.649**	10.342	16.401	16.329	12.749	**16.384**	7.357	14.844	16.257	**22.978**
**pic11**	**20.233**	10.832	15.117	15.399	18.494	20.081	8.386	**21.681**	19.094	**20.562**
**pic12**	**18.292**	7.383	6.569	16.037	17.283	14.765	13.705	**20.402**	16.716	**20.018**
**pic13**	**19.407**	9.600	15.969	16.923	12.082	**18.322**	8.620	15.642	16.364	**22.060**
**pic14**	**22.080**	9.758	9.744	16.778	16.677	18.019	12.688	**20.009**	18.477	**25.291**
**pic15**	**19.098**	9.377	8.097	14.005	**18.146**	16.901	11.593	15.848	14.165	**20.544**
**pic16**	**18.317**	12.510	**18.999**	13.508	11.512	17.790	7.151	13.618	13.849	**24.290**
**pic17**	**22.489**	9.448	**21.741**	15.868	14.099	17.275	8.407	16.287	17.540	**23.411**
**pic18**	**23.664**	10.217	15.224	14.615	12.703	**17.613**	9.708	16.968	15.447	**26.951**
**pic19**	**20.465**	9.651	**20.935**	16.003	13.337	18.303	8.521	16.300	15.838	**21.191**
**pic20**	**18.721**	12.028	9.209	15.156	17.349	**20.096**	10.723	16.869	14.501	**21.910**

**Table 4:** Average PSNR of two groups of images of MIT-5K.

PSNR	AGCWD	Al-Ameen	BHE2PL	BIMEF	BPDHE	LIME	MSRCR	NPEA	SRIE	OURS
**20 random images of MIT-5K data set**	**20.790**	10.314	13.273	15.602	15.875	**17.464**	10.233	17.337	16.140	**22.417**
**Top 100 images of MIT-5K data set**	**20.733**	9.670	12.502	17.417	**19.117**	15.973	12.379	17.197	18.367	**20.034**

#### 4.3.2 Image structural similarity (SSIM)

In addition to analyzing the differences between input and output images from a mathematical point of view, some researchers found that natural images show some special structural features, such as strong correlations between pixels, which capture most of the important structural information for the image. Therefore, Wang et al. [49] proposed an image quality evaluation method based on structural similarity (SSIM), SSIM evaluates the quality of the processed image relative to the reference image according to the comparison of brightness *l*(*x*,*y*), contrast *c*(*x*,*y*) and structure *s*(*x*,*y*) between the two images. The three values are combined to obtain the overall similarity measure *SSIM*(*x*,*y*).


SSIM(x,y)=f(l(x,y)⋅c(x,y)⋅s(x,y)) (47)

From the value of SSIM in Table 5, the average SSIM obtained by the method in this paper is the highest in 20 random images of MIT-5K, followed by LIME and AGCWD, NPEA ranked fourth. On the whole, our method has an absolute advantage. Because only one image of 20 random images of MIT-5K ranks sixth in SSIM value, and the SSIM values of other images of 20 random images of MIT-5K are in the top three. In addition, as show in Table 6, the average SSIM of the top 100 images of MIT-5K is rank second, AGCWD ranks first here and SRIE ranks third. It shows that the method in this paper can maintain the image structure well.

**Table 5:** SSIM values of 20 random images of MIT-5K.

SSIM	AGCWD	Al-Ameen	BHE2PL	BIMEF	BPDHE	LIME	MSRCR	NPEA	SRIE	OURS
**pic1**	**0.928**	0.687	0.610	0.847	0.857	0.869	0.317	**0.909**	0.899	**0.901**
**pic2**	**0.837**	0.595	0.428	0.678	0.760	**0.800**	0.414	0.772	0.768	**0.849**
**pic3**	**0.839**	0.562	0.310	0.554	**0.765**	0.696	0.151	0.696	0.638	**0.859**
**pic4**	**0.710**	0.647	0.338	0.550	0.662	**0.725**	0.451	0.679	0.603	**0.749**
**pic5**	0.760	0.619	0.848	0.766	0.515	**0.915**	0.146	**0.862**	0.821	**0.880**
**pic6**	**0.854**	0.422	0.659	0.701	0.803	**0.798**	0.480	0.779	0.795	**0.824**
**pic7**	0.766	0.468	0.347	0.586	0.691	**0.803**	0.577	**0.826**	0.722	**0.917**
**pic8**	**0.806**	0.516	**0.812**	0.753	0.673	0.778	0.284	0.779	0.792	**0.818**
**pic9**	0.728	0.738	0.755	0.664	0.740	**0.792**	0.476	**0.835**	0.744	**0.861**
**pic10**	**0.793**	0.473	0.716	0.641	0.633	**0.783**	0.224	0.736	0.718	**0.806**
**pic11**	0.821	0.729	0.826	0.783	0.783	**0.854**	0.295	**0.848**	**0.843**	0.814
**pic12**	0.648	0.344	0.360	0.488	0.625	**0.692**	0.216	**0.814**	0.614	**0.869**
**pic13**	0.748	0.519	**0.785**	0.611	0.559	**0.784**	0.348	0.661	0.675	**0.804**
**pic14**	0.801	0.572	0.578	0.701	0.740	**0.844**	0.567	**0.817**	0.797	**0.872**
**pic15**	**0.706**	0.511	0.484	0.541	0.672	**0.703**	0.536	0.676	0.618	**0.752**
**pic16**	0.717	0.642	**0.802**	0.688	0.585	0.742	0.199	0.694	**0.735**	**0.791**
**pic17**	**0.787**	0.590	**0.778**	0.692	0.682	0.767	0.336	0.726	0.748	**0.794**
**pic18**	**0.886**	0.631	0.835	0.792	0.648	**0.874**	0.380	0.848	0.849	**0.889**
**pic19**	0.728	0.500	**0.735**	0.569	0.528	**0.749**	0.297	0.711	0.661	**0.772**
**pic20**	0.734	0.565	0.415	0.579	0.708	**0.779**	0.401	**0.740**	0.679	**0.805**

**Table 6:** Average SSIM of two groups of images of MIT-5K.

SSIM	AGCWD	Al-Ameen	BHE2PL	BIMEF	BPDHE	LIME	MSRCR	NPEA	SRIE	OURS
**20 random images of MIT-5K data set**	**0.780**	0.566	0.621	0.659	0.681	**0.787**	0.355	0.770	0.736	**0.831**
**Top 100 images of MIT-5K data set**	**0.815**	0.549	0.591	0.720	0.753	0.764	0.398	0.767	**0.794**	**0.804**

#### 4.3.3 Image information entropy (entropy)

The information entropy of an image represents the average amount of information conveyed by each gray-level pixel of the image, which is used to measure the importance of the target in the image. The larger the value, the richer the image details and the better the image quality. The calculation method of image information entropy is Formula (48). As shown in Tables 7 and 8, it is the information entropy value of the image processed by each method. From the data in the table, where the method in this paper has achieved outstanding results in the 20 random images of MIT-5K, the entropy value of each image here is excellent, and the average value of the method in this paper is the highest in the three group of images. In the group of 20 random images of MIT-5K, the method in this paper ranked first, AGCWD was the second and BPDHE was the third; In the group of top 100 images of MIT-5K, AGCWD was the first, the method in this paper was the second and LIME was the third; In the group of DICM data set, the method in this paper ranked first, BPDHE was the second and NPEA was the third; It shows that the method proposed in this paper shows considerable advantages in increasing the amount of image information.


$$H=-\sum_{i=0}^{255}p(i)\times \log_{2}(p(i))$$ (48)

**Table 7:** Image information entropy values of 20 random images of MIT-5K.

Entropy	AGCWD	Al-Ameen	BHE2PL	BIMEF	BPDHE	LIME	MSRCR	NPEA	SRIE	OURS
**pic1**	**7.393**	7.369	5.152	**7.513**	7.231	7.534	6.327	**7.411**	7.270	**7.380**
**pic2**	**7.829**	7.580	6.034	7.076	7.725	**7.875**	6.955	7.512	7.204	**7.852**
**pic3**	**7.745**	6.814	4.137	6.025	**7.549**	6.912	6.784	7.189	6.192	**7.804**
**pic4**	7.655	**7.779**	6.274	6.958	7.547	**7.762**	6.936	7.742	7.045	**7.876**
**pic5**	**7.775**	6.943	7.528	7.117	7.303	**7.539**	6.798	7.091	7.342	**7.740**
**pic6**	**7.915**	6.973	7.104	7.108	**7.660**	**7.752**	7.160	7.431	7.346	**7.473**
**pic7**	**7.783**	6.587	4.821	6.431	**7.755**	7.222	7.633	7.196	6.747	**7.916**
**pic8**	**7.688**	6.542	6.912	7.345	**7.588**	7.551	6.751	7.237	7.417	**7.753**
**pic9**	**7.716**	6.532	4.882	6.244	**7.727**	6.850	7.655	7.271	6.395	**7.616**
**pic10**	**7.756**	6.940	7.306	7.038	**7.454**	7.515	6.101	6.975	7.278	**7.867**
**pic11**	**7.797**	6.048	5.376	6.867	**7.940**	7.453	7.329	7.397	7.038	**7.863**
**pic12**	**7.694**	6.297	5.395	6.329	**7.809**	7.098	7.604	7.312	6.549	**7.848**
**pic13**	**7.773**	6.461	7.210	7.063	**7.419**	7.466	7.145	7.079	7.340	**7.752**
**pic14**	**7.812**	6.895	5.609	6.946	**7.630**	7.627	7.590	7.249	7.172	**7.857**
**pic15**	**7.865**	7.078	5.433	6.831	**7.838**	7.677	7.442	7.430	6.995	**7.901**
**pic16**	**7.679**	6.859	6.230	7.020	7.452	**7.718**	6.233	7.272	7.200	**7.626**
**pic17**	**7.903**	7.008	7.476	7.122	7.523	**7.748**	6.565	7.237	7.350	**7.952**
**pic18**	**7.915**	6.743	7.193	7.100	7.637	**7.680**	7.396	7.417	7.330	**7.958**
**pic19**	**7.848**	7.057	**7.623**	6.867	7.394	7.601	6.418	7.099	7.098	**7.906**
**pic20**	**7.859**	7.220	5.316	6.842	**7.687**	7.646	7.271	7.254	7.042	**7.929**

**Table 8:** Average Entropy of three groups of images.

Entropy	AGCWD	Al-Ameen	BHE2PL	BIMEF	BPDHE	LIME	MSRCR	NPEA	SRIE	OURS
**20 random images of MIT-5K data set**	**7.770**	6.886	6.151	6.892	**7.593**	7.511	7.005	7.290	7.068	**7.793**
**Top 100 images of MIT-5K data set**	**7.691**	7.063	6.388	6.895	7.481	**7.502**	6.550	7.317	7.056	**7.589**
**DICM data set**	7.215	6.269	6.517	7.323	**7.375**	7.247	5.417	**7.366**	7.213	**7.453**

#### 4.3.4 Image average gradient (AG)

The average gradient of an image refers to the average value of all pixels on an image gradient map, which reflects the characteristics of detailed texture changes of the image, and the clarity of the image. The larger the average gradient value, the richer the image level and the clearer the image. The calculation formula of the average gradient AG is as follows:

$$AG=\frac{1}{M\times N}\sum_{i=1}^{M}\sum_{j=1}^{N}\sqrt{\frac{{\left(\frac{\partial f}{\partial x}\right)}^{2}+{\left(\frac{\partial f}{\partial y}\right)}^{2}}{2}}$$ (49)

Where *M*×*N* represents the image size, $\frac{\partial f}{\partial x}$ and $\frac{\partial f}{\partial y}$ represent the gradient in the horizontal and vertical directions respectively.

As shown in Table 9, it is the AG value of the 20 random images of MIT-5K processed by each method. From the data in the Table 9, most of the AG values obtained from the images processed by the method in this paper are ranked in the top three, only one image is ranked third with BHE2PL, and one image is ranked fourth. In addition, as shown in Table 10, in the group of 20 random images of MIT-5K, the average AG value of the method in this paper ranked first, LIME was the second and AGCWD was the third; In the group of top 100 images of MIT-5K, LIME was the first, the method in this paper was the second and AGCWD was the third; In the group of DICM data set, LIME was the first, the method in this paper was the second and BHE2PL was the third. The method in this paper performs well on three groups of experimental images, indicating that the method in this paper has outstanding performance in improving image clarity.

**Table 9:** Image average gradient values of 20 random images of MIT-5K.

AG	AGCWD	Al-Ameen	BHE2PL	BIMEF	BPDHE	LIME	MSRCR	NPEA	SRIE	OURS
**pic1**	6.084	**7.195**	**7.662**	5.445	5.338	**8.269**	3.686	5.746	5.486	**7.163**
**pic2**	9.372	8.547	**10.201**	5.934	8.846	**11.381**	7.964	8.905	7.053	**10.242**
**pic3**	**6.343**	3.357	2.897	2.063	6.005	4.274	3.508	**6.917**	2.693	**7.370**
**pic4**	6.185	6.989	**8.869**	3.992	5.410	**7.431**	4.689	6.864	4.166	**7.526**
**pic5**	**3.719**	3.506	**4.166**	2.754	2.663	4.371	2.099	3.281	3.169	**4.197**
**pic6**	**5.610**	5.256	3.919	4.159	4.660	**7.447**	3.838	5.107	4.816	**7.244**
**pic7**	5.537	3.145	4.595	2.399	**6.755**	4.373	**5.752**	3.681	2.941	**6.407**
**pic8**	**7.110**	4.759	6.749	5.693	5.585	**9.057**	3.938	5.917	6.202	**7.802**
**pic9**	4.715	4.449	4.738	2.278	**5.445**	4.020	**5.404**	4.889	2.889	**5.959**
**pic10**	5.870	4.041	5.142	3.529	**6.078**	**6.663**	2.456	5.463	4.176	**6.301**
**pic11**	3.406	2.790	**3.906**	2.244	**3.479**	**4.113**	2.674	3.359	2.904	**3.906**
**pic12**	8.575	3.433	4.773	3.419	**9.812**	6.187	**13.321**	7.040	4.208	**11.049**
**pic13**	**9.444**	4.495	8.347	5.293	7.245	**8.910**	5.864	6.071	6.163	**9.512**
**pic14**	6.234	4.018	6.154	3.383	5.212	**6.389**	**6.688**	4.652	4.328	**6.815**
**pic15**	**5.528**	3.768	4.737	2.733	**5.615**	4.961	5.437	4.339	3.303	**6.601**
**pic16**	**6.309**	4.449	5.581	4.284	5.381	**8.036**	4.064	6.223	4.920	**7.012**
**pic17**	**5.339**	3.571	4.760	3.139	3.905	**5.829**	2.467	4.316	3.935	**6.207**
**pic18**	**9.205**	6.875	5.974	5.531	**7.852**	10.117	5.660	7.052	6.497	**10.546**
**pic19**	**8.786**	4.994	6.672	4.268	7.264	**7.622**	3.655	6.471	5.097	**10.033**
**pic20**	**5.609**	3.895	4.559	2.750	4.809	**5.041**	4.851	4.272	3.273	**6.065**

**Table 10:** Average image average gradient values of three groups of images.

AG	AGCWD	Al-Ameen	BHE2PL	BIMEF	BPDHE	LIME	MSRCR	NPEA	SRIE	OURS
**20 random images of** **MIT-5K data set**	**6.449**	4.676	5.720	3.764	5.868	**6.724**	4.901	5.528	4.411	**7.398**
**Top 100 images of** **MIT-5K data set**	**6.984**	5.114	5.996	4.268	6.322	**7.475**	4.641	6.054	4.880	**7.195**
**DICM data set**	5.750	5.891	**7.318**	5.885	5.690	**8.371**	2.969	6.579	5.579	**7.455**

#### 4.3.5 Image contrast ratio (CR)

Contrast ratio refers to the measurement of different brightness levels between the brightest white and the darkest black in an image. The larger the difference range, the greater the contrast ratio, and the smaller the difference range, the smaller the contrast ratio. Generally speaking, the higher the contrast ratio, the clearer and more striking the image, and the brighter the colors, while the lower the contrast, the whole picture will be gray. The calculation formula of image contrast is as follows:

$$CR=\sum_{\delta }\delta {\left(i,j\right)}^{2}P_{\delta }(i,j)$$ (50)

Where *δ*(*i*,*j*) = |*i*−*j*| is the gray level difference between adjacent pixels, and *P*_*δ*_(*i*,*j*) is the probability distribution of the pixel whose gray level difference is *δ* between adjacent pixels. As shown in Table 11, it is the contrast value of the 20 random images of MIT-5K processed by each method. Among all the test images, the image processed by the method proposed in this paper has nine images that ranked first in contrast ratio, seven images ranked second, three images ranked third, and only one ranked fourth. Among them, Al-Ameen, BHE2PL, LIME and MSRCR method occasionally have a better contrast than the method in this paper. However, from the overall average of Table 12, only LIME has surpassed the method in the last two groups of image experiments. Besides, the method is the best. The method in this paper is relatively advantageous in contrast enhancement, although not the best performer in all images, the overall ranking is high. At the same time, we can draw some reasons from the image visual perception in the previous section. The occasional lack of contrast in our method is to maintain a better visual effect while retaining a more natural appearance and image details.

**Table 11:** Image contrast ratio values of 20 random images of MIT-5K.

CR	AGCWD	Al-Ameen	BHE2PL	BIMEF	BPDHE	LIME	MSRCR	NPEA	SRIE	OURS
**pic1**	134.173	**208.039**	**332.810**	95.136	90.031	**250.145**	53.509	106.158	98.541	196.560
**pic2**	270.253	241.423	**474.457**	95.757	253.451	**372.712**	275.492	216.547	134.559	**309.588**
**pic3**	**179.182**	51.412	68.995	17.396	177.768	77.980	52.242	**188.250**	28.858	**259.207**
**pic4**	189.490	235.788	**472.179**	71.904	131.144	**267.031**	124.562	201.927	77.950	**273.905**
**pic5**	87.769	91.907	**115.036**	47.011	45.123	**117.639**	29.328	62.421	61.453	**109.633**
**pic6**	**180.586**	169.329	86.252	94.076	117.678	**310.385**	89.694	136.068	126.592	**349.665**
**pic7**	**178.853**	85.230	202.281	45.668	**246.500**	154.601	165.409	97.995	64.897	**317.198**
**pic8**	**263.401**	141.072	256.308	180.053	177.067	**412.557**	134.963	177.319	206.484	**317.343**
**pic9**	104.817	112.683	**147.365**	28.735	**139.824**	93.010	104.119	126.797	48.122	**191.564**
**pic10**	**245.936**	160.159	239.990	95.374	238.703	**335.635**	57.411	202.191	130.228	**285.792**
**pic11**	68.677	59.145	**118.389**	30.129	56.017	**104.480**	31.849	62.422	51.274	**80.986**
**pic12**	274.346	90.183	180.112	48.150	**373.442**	147.744	**640.117**	184.517	71.621	**460.509**
**pic13**	**376.866**	100.447	298.765	133.116	234.299	**335.479**	182.807	164.370	173.102	**357.201**
**pic14**	**172.027**	76.849	**203.695**	46.899	113.132	167.299	167.577	86.698	76.656	**193.035**
**pic15**	107.517	58.101	**116.484**	24.093	**114.198**	77.223	91.579	56.857	35.377	**161.384**
**pic16**	203.458	125.929	**237.409**	95.161	137.895	**326.603**	113.267	157.644	123.354	**266.446**
**pic17**	**129.151**	71.260	108.199	43.640	69.883	**144.718**	30.741	72.964	66.617	**167.846**
**pic18**	**412.579**	255.022	176.199	151.890	290.490	**502.766**	169.054	232.147	207.042	**545.416**
**pic19**	**300.079**	124.523	182.543	84.327	**246.517**	227.285	62.213	168.725	111.032	**409.665**
**pic20**	**172.806**	100.640	**185.382**	41.572	117.099	137.642	116.637	88.249	57.432	**198.325**

**Table 12:** Average image contrast ratio values of three groups of images.

CR	AGCWD	Al-Ameen	BHE2PL	BIMEF	BPDHE	LIME	MSRCR	NPEA	SRIE	OURS
**20 random images of MIT-5K data set**	**202.60**	127.96	**210.14**	73.50	168.51	228.15	134.63	139.51	97.56	**272.56**
**Top 100 images of MIT-5K data set**	**246.41**	134.61	200.99	97.24	204.98	**283.85**	131.38	159.41	119.35	**262.29**
**DICM**	205.75	259.95	**374.24**	193.26	174.56	**413.15**	70.30	219.02	175.20	**317.26**

#### 4.3.6 Image brightness difference (BD)

The average value of the image reflects the brightness of the image. The larger the average value, the greater the brightness of the image, and vice versa. Its value can be calculated by the formula:

$$MEAN=\frac{\sum_{y=1}^{M}\sum_{x=1}^{N}g(x,y)}{M\times N}$$ (51)

Where M×N is the image size, which is the gray value of the pixel in the x row and y column of the image. Under normal circumstances, brightness is an important indicator in evaluating the quality of an image. An image needs sufficient brightness to bring people a good visual experience, but it is not that the larger the brightness value, the better the visual effect. The reference [50] pointed out that when the image gray value is around 128, it indicates that the visual effect is good. In Formula (52), BD represents the difference between the average brightness of the test image and 128, the smaller the value of BD, the better the visual effect of the test image enhancement result. Its definition is as follows:

BD=|MEAN−128| (52)

As shown in Table 13, it is the brightness difference value of the 20 random images of MIT-5K processed by each method. Among all the test images, most of the images processed by the method proposed in this paper are close to 128 in brightness difference, and from the average value of the three groups of images in Table 14, the method proposed in this paper is relatively stable in maintaining the visual comfort brightness of the image, it maintains consistent performance on low-illuminance images in different environments. NPEA is inferior to the method proposed in this paper, and the performance of AGCWD, BHE2PL and LIME is not stable. Although they show good results in a single image, these three methods are not adaptable, and no consistent good results are shown in other low light images.

**Table 13:** Image brightness difference values of 20 random images of MIT-5K.

BD	AGCWD	Al-Ameen	BHE2PL	BIMEF	BPDHE	LIME	MSRCR	NPEA	SRIE	OURS
**pic1**	44.095	**26.435**	44.417	**33.239**	57.385	**21.670**	90.502	42.096	48.542	45.955
**pic2**	28.750	39.265	58.202	25.728	41.561	**1.774**	73.097	**18.750**	42.877	**21.019**
**pic3**	**15.674**	47.635	111.178	12.774	34.268	**16.522**	80.288	**13.898**	21.510	17.171
**pic4**	47.489	**14.278**	59.029	41.697	51.964	22.746	79.778	**12.424**	63.824	**19.575**
**pic5**	21.439	75.363	31.029	15.003	60.591	**12.131**	84.476	**13.100**	23.303	**4.376**
**pic6**	**10.723**	75.408	28.169	12.842	30.832	16.437	73.159	**6.731**	22.640	**4.578**
**pic7**	**1.207**	78.148	88.724	**5.144**	15.226	19.951	38.955	12.615	15.568	**7.611**
**pic8**	**4.297**	85.016	7.895	**1.861**	36.863	32.461	79.261	**5.005**	11.785	8.768
**pic9**	**6.356**	71.581	80.650	**5.265**	17.919	26.260	36.377	30.037	**7.742**	22.724
**pic10**	19.237	70.832	33.501	**16.627**	49.732	17.638	96.381	**13.020**	21.351	**4.723**
**pic11**	15.278	90.520	66.576	8.599	**6.113**	41.422	62.026	34.586	**6.251**	**13.406**
**pic12**	**5.518**	88.342	101.247	9.045	**7.755**	23.525	31.897	**8.598**	9.435	13.827
**pic13**	**3.242**	88.157	49.365	**0.834**	42.118	34.807	71.604	11.019	10.029	**6.469**
**pic14**	**9.666**	79.678	81.550	20.467	31.615	26.468	47.977	**3.869**	14.847	**2.777**
**pic15**	17.515	68.725	86.144	15.791	17.881	14.077	57.631	**8.688**	25.362	**11.138**
**pic16**	16.130	59.789	33.101	26.566	48.923	15.261	88.667	6.568	29.396	**7.268**
**pic17**	13.360	73.342	**2.882**	26.392	47.116	22.989	90.378	**1.950**	19.697	**4.576**
**pic18**	**1.092**	77.652	21.383	15.379	39.423	24.582	62.764	**2.638**	20.118	**8.883**
**pic19**	21.829	65.453	**7.617**	19.904	55.297	7.984	94.278	**0.396**	32.957	**9.343**
**pic20**	20.256	52.887	78.140	21.506	28.688	**2.551**	63.038	**6.870**	35.059	**5.796**

**Table 14:** Average image brightness difference values of three groups of images.

BD	AGCWD	Al-Ameen	BHE2PL	BIMEF	BPDHE	LIME	MSRCR	NPEA	SRIE	OURS
**20 random images of MIT-5K data set**	**16.158**	66.425	53.540	16.733	36.064	20.063	70.127	**12.643**	24.115	**11.999**
**Top 100 images of MIT-5K data set**	25.671	59.087	49.663	23.984	45.027	**15.893**	79.230	**16.859**	33.693	**23.660**
**DICM data set**	38.110	68.576	**29.264**	35.582	44.370	37.745	89.450	**34.082**	41.028	**26.615**

#### 4.3.7 Image standard deviation (STD)

The image gray standard deviation reflects the degree of dispersion between the image pixel value and the image average. The larger the standard deviation, the better the image quality. The calculation formula of the image gray standard deviation is as follows:

$$STD=\sqrt{\frac{1}{M\times N}\sum_{x=1}^{M}\sum_{y=1}^{N}{\left(g(x,y)-MEAN\right)}^{2}}$$ (53)

**Fig 31:**
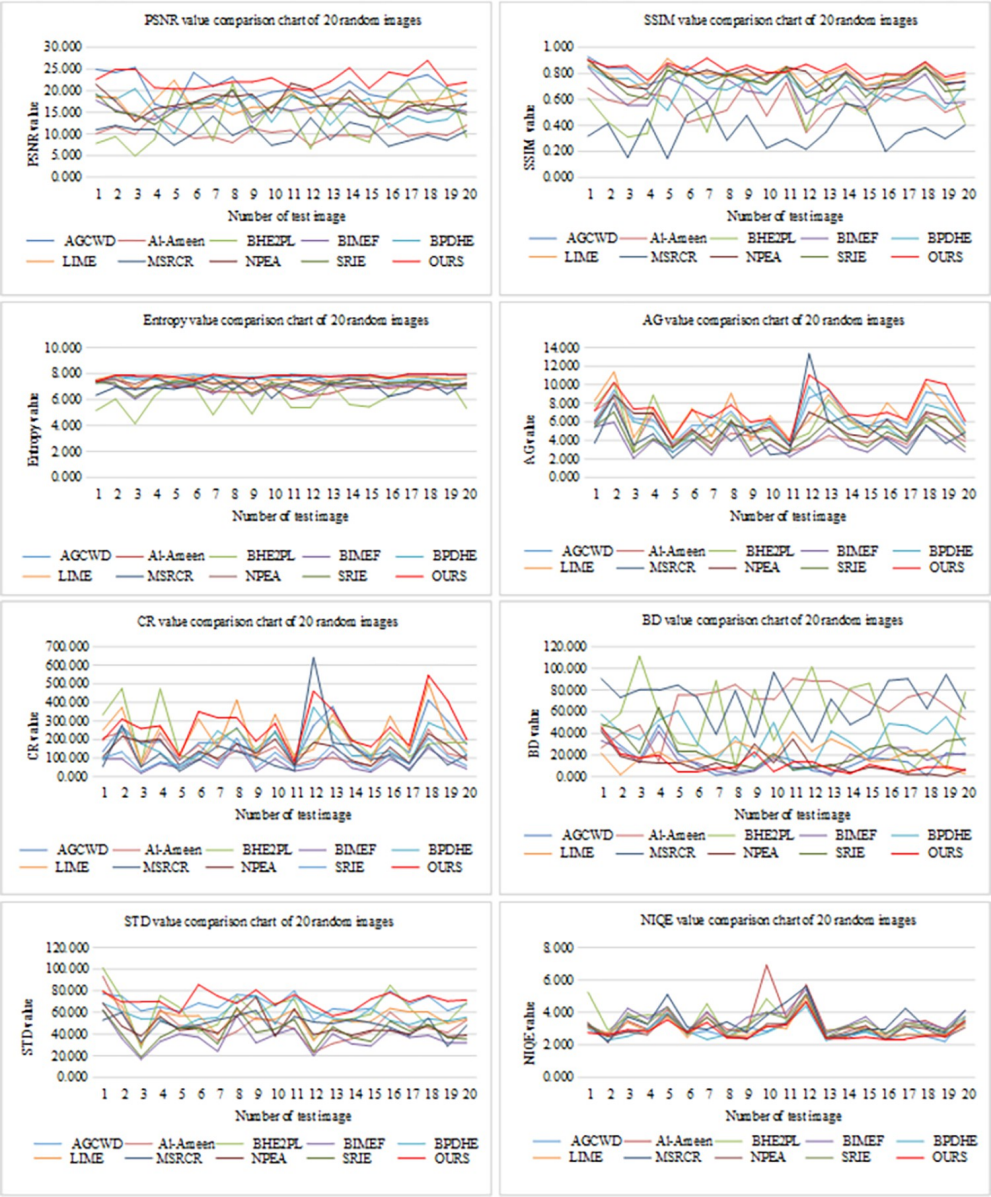
20 random images with different method score line graphs for each index value.

As shown in Table 15, it is the standard deviation value of the 20 random images of MIT-5K processed by each method. Among all the test images, the method proposed in this article has achieved the first ranking with nine test images, six test images ranked second, two test images ranked third, and two images ranked fourth, and it can be seen more intuitively from Fig 31 that although the STD value of the image processed by the proposed method is not absolutely optimal, its performance is better overall. In addition, as shown in Table 16, the method proposed in this paper and Al-Ameen’s method and BHE2PL show good performance on STD data. Among the three groups of test images, Al-Ameen’s method ranks second, first and third respectively, BHE2PL ranks third, third and first respectively, while our method ranks first, second and second respectively. Compared with other methods in this paper, our method is relatively stable on low-light images in different environments, which shows that the method proposed in this paper also has a certain advantage in improving image quality.

**Table 15:** Image standard deviation values of 20 random images of MIT-5K.

STD	AGCWD	Al-Ameen	BHE2PL	BIMEF	BPDHE	LIME	MSRCR	NPEA	SRIE	OURS
**pic1**	77.196	**93.269**	**101.057**	61.965	68.488	**80.081**	52.807	68.479	62.307	79.399
**pic2**	**74.777**	59.198	**72.947**	35.892	60.786	65.635	59.825	47.263	39.918	**69.473**
**pic3**	**61.413**	30.416	27.216	16.545	**54.064**	30.298	32.562	38.050	18.723	**69.930**
**pic4**	**65.072**	62.724	**75.388**	33.066	53.584	60.708	52.127	56.070	36.901	**69.495**
**pic5**	**60.887**	47.656	**64.568**	40.013	45.501	56.696	45.405	43.424	45.815	**59.138**
**pic6**	**68.573**	48.570	42.204	36.984	53.594	**57.233**	48.260	45.453	46.033	**85.582**
**pic7**	**64.202**	34.246	48.847	23.959	**55.222**	39.404	53.073	40.865	30.891	**75.016**
**pic8**	**76.401**	42.406	**74.944**	56.979	**70.078**	61.830	56.923	56.321	64.355	68.319
**pic9**	**75.096**	55.018	57.932	31.850	**75.436**	53.313	61.634	74.026	41.502	**80.734**
**pic10**	**65.755**	51.432	**68.247**	39.654	49.076	53.641	39.012	37.994	44.739	**67.119**
**pic11**	**79.923**	44.762	71.993	45.142	**74.546**	62.630	56.159	63.115	53.385	**76.247**
**pic12**	**52.955**	23.229	35.248	19.993	**60.390**	34.007	51.064	39.460	23.596	**66.249**
**pic13**	**63.395**	31.278	52.777	40.112	**54.681**	52.237	49.766	43.808	46.818	**56.740**
**pic14**	**61.875**	35.644	**54.064**	31.023	51.410	51.305	53.461	38.828	36.946	**60.703**
**pic15**	**63.635**	42.301	58.443	28.950	**63.488**	51.950	50.426	43.338	33.206	**72.364**
**pic16**	**79.933**	60.242	85.105	44.315	52.468	**63.779**	46.240	43.123	51.955	**78.346**
**pic17**	**67.409**	46.358	**64.558**	36.882	48.754	60.406	38.520	40.103	42.871	**69.842**
**pic18**	**74.952**	45.460	47.102	38.767	54.005	**59.867**	54.655	48.527	48.721	**75.503**
**pic19**	**61.637**	41.455	51.154	31.717	**52.081**	49.783	28.871	37.759	36.672	**70.375**
**pic20**	**67.859**	52.781	**69.528**	31.074	55.498	53.674	48.158	38.934	35.429	**71.518**

**Table 16:** Average image standard deviation values of three groups of images.

STD	AGCWD	Al-Ameen	BHE2PL	BIMEF	BPDHE	LIME	MSRCR	NPEA	SRIE	OURS
**20 random images of MIT-5K data set**	**68.147**	47.422	**61.166**	36.244	57.658	54.924	48.947	47.247	42.039	**71.105**
**Top 100 images of MIT-5K data set**	**65.983**	47.289	**56.231**	36.269	54.490	55.261	45.623	45.598	40.730	**61.717**
**DICM data set**	**74.168**	65.811	**82.874**	60.660	63.620	72.448	43.556	61.871	63.303	**77.328**

#### 4.3.8 Natural image quality evaluator (NIQE)

After evaluating the experimental images based on the above evaluation indexes, we also use NIQE to evaluate the experimental images. NIQE is based on a set of “quality perception” features and fits them into the MVG model. The quality perception feature comes from a simple but highly regularized NSS model. Then, the NIQE index of the given test image is expressed as the distance between the MVG model of NSS features extracted from the test image and the MVG model of quality perception features extracted from natural image corpus [51]. Its image evaluation results are more consistent with the feeling of human eyes observing the image, and the smaller the value of NIQE, the better the visual effect of the image.

As shown in Table 17, it is the NIQE value of the 20 random images of MIT-5K processed by each method. Among all the test images, most of the images processed by the method in this paper can get relatively good NIQE values, and on the whole, as shown in Table 18, the NIQE value of the method in this paper is also very good in the three groups of test images, and the average value of the three groups images of the method in this paper is the smallest. It shows that the method in this paper has obtained good visual effects in image processing.

**Table 17:** NIQE values of 20 random images of MIT-5K.

NIQE	AGCWD	Al-Ameen	BHE2PL	BIMEF	BPDHE	LIME	MSRCR	NPEA	SRIE	OURS
**pic1**	3.107	3.389	5.249	3.228	**3.016**	**3.078**	3.280	3.173	3.291	**2.749**
**pic2**	2.525	2.164	2.914	2.662	2.297	2.743	**2.175**	**2.486**	**2.510**	2.623
**pic3**	**2.852**	3.475	3.693	4.259	**2.529**	3.389	3.735	**2.799**	3.943	2.928
**pic4**	2.946	2.959	3.831	3.600	2.980	**2.821**	3.267	**2.632**	3.304	**2.821**
**pic5**	3.866	4.161	3.846	4.343	3.978	**3.736**	5.092	**3.861**	4.305	**3.540**
**pic6**	**2.664**	2.744	2.646	2.820	2.842	**2.455**	3.097	2.839	**2.636**	2.756
**pic7**	**2.847**	3.956	4.531	4.034	**2.336**	3.710	**2.955**	3.695	3.712	3.380
**pic8**	**2.519**	2.947	**2.497**	2.741	2.634	2.566	3.416	2.529	2.770	**2.432**
**pic9**	3.118	2.794	2.965	3.691	**2.411**	2.607	2.780	**2.355**	3.160	**2.388**
**pic10**	**2.823**	6.912	4.832	3.977	**2.760**	3.168	3.816	3.284	4.002	**3.120**
**pic11**	3.409	3.686	3.489	4.037	**3.299**	**3.003**	4.642	3.313	3.642	**3.234**
**pic12**	**4.591**	5.705	**4.633**	5.408	**4.376**	4.952	5.588	5.119	5.127	4.667
**pic13**	2.485	2.880	2.802	2.695	**2.272**	2.514	2.508	**2.436**	2.447	**2.392**
**pic14**	**2.401**	3.012	3.174	3.152	2.641	**2.481**	2.575	2.780	3.088	**2.380**
**pic15**	**2.875**	3.107	2.917	3.736	**2.768**	3.147	2.951	3.180	3.489	**2.474**
**pic16**	2.342	**2.318**	2.685	2.644	2.431	2.524	2.998	**2.311**	2.715	**2.327**
**pic17**	**2.639**	3.295	3.112	3.576	3.155	**2.636**	4.251	3.152	3.355	**2.373**
**pic18**	**2.532**	3.488	3.246	3.339	**2.597**	2.638	3.090	2.999	3.169	**2.556**
**pic19**	**2.203**	2.949	2.571	2.995	2.678	2.639	2.753	**2.463**	2.857	**2.510**
**pic20**	3.665	3.426	3.546	4.131	**3.322**	**3.249**	4.098	**3.073**	3.754	3.472

**Table 18:** Average NIQE values of three groups of images.

NIQE	AGCWD	Al-Ameen	BHE2PL	BIMEF	BPDHE	LIME	MSRCR	NPEA	SRIE	OURS
**20 random images of MIT-5K data set**	**2.920**	3.468	3.459	3.553	**2.866**	3.003	3.453	3.024	3.364	**2.856**
**Top 100 images of MIT-5K data set**	**2.933**	3.200	3.640	3.498	**2.930**	3.005	3.493	2.973	3.356	**2.900**

## 5 Conclusion

In this paper, a low-light image enhancement algorithm with brightness equalization and detail preservation is proposed. The image is processed in both directions. On the one hand, the proposed dual-histogram dual-automatic platform equalization method based on improved CS algorithm is used to improve image brightness and contrast. On the other hand, the image detail mask is made by the method based on the total variation model. Finally, the results of the two parties are merged to obtain the final enhanced image. The main contributions of this paper are: (1) The method in this paper can maintain image detail information while equalizing image brightness and improving image contrast; (2) This paper proposes a new search optimization strategy based on CS and PSO, which is not easy to fall into locally optimal, and can maintain the exploration ability in the later stage, which is more conducive to the selection of the optimal value. Through experiments, from the results of human subjective evaluation and objective index evaluation, it is shown that this method has a good effect in processing low-light images, and this method is suitable for low-light images produced in various environments.

## Supporting information

S1 File (7Z)

S1 Data (XLSX)
